# Potency and durability of T and B cell immune responses after homologous and heterologous vector delivery of a trimer-stabilized, membrane-displayed HIV-1 clade ConC Env protein

**DOI:** 10.3389/fimmu.2023.1270908

**Published:** 2023-11-17

**Authors:** Beatriz Perdiguero, Alexandra Hauser, Carmen Elena Gómez, David Peterhoff, Elefthéria Sideris, Carlos Óscar S. Sorzano, Sarah Wilmschen, Marion Schaber, Laura Stengel, Benedikt Asbach, Song Ding, Dorothee Von Laer, Yves Levy, Giuseppe Pantaleo, Janine Kimpel, Mariano Esteban, Ralf Wagner

**Affiliations:** ^1^ Department of Molecular and Cellular Biology, Centro Nacional de Biotecnología, Consejo Superior de Investigaciones Científicas, Madrid, Spain; ^2^ Centro de Investigación Biomédica en Red de Enfermedades Infecciosas (CIBERINFEC), Instituto de Salud Carlos III (ISCIII), Madrid, Spain; ^3^ Institute of Medical Microbiology and Hygiene, University of Regensburg, Regensburg, Germany; ^4^ Biocomputing Unit and Computational Genomics, Centro Nacional de Biotecnología, Consejo Superior de Investigaciones Científicas, Madrid, Spain; ^5^ Institute of Virology, Medical University of Innsbruck, Innsbruck, Austria; ^6^ EuroVacc Foundation, Lausanne, Switzerland; ^7^ Vaccine Research Institute (VRI), Université Paris-Est Créteil, Faculté de Médicine, Institut national de la santé et de la recherche médicale (INSERM) U955, Créteil, France; ^8^ Institut national de la santé et de la recherche médicale (INSERM) U955, Equipe 16, Créteil, France; ^9^ Assistance Publique-Hôpitaux de Paris (AP-HP), Hôpital Henri-Mondor Albert-Chenevier, Service d'Immunologie Clinique et Maladies Infectieuses, Créteil, France; ^10^ Division of Immunology and Allergy, Department of Medicine, Centre Hospitalier Universitaire Vaudois and University of Lausanne, Lausanne, Switzerland; ^11^ Institute of Clinical Microbiology and Hygiene, University Hospital Regensburg, Regensburg, Germany

**Keywords:** trimeric ConCv5 KIKO protein, HIV-1 vaccine, DNA, VSV-GP and NYVAC vectors, membrane display, mice immunization, T and B cells, antibodies

## Abstract

**Introduction:**

The generation of an HIV-1 vaccine able to induce long-lasting protective immunity remains a main challenge. Here, we aimed to modify next-generation soluble, prefusion-stabilized, close-to-native, glycan-engineered clade C gp140 envelope (Env) trimers (sC23v4 KIKO and ConCv5 KIKO) for optimal display on the cell surface following homologous or heterologous vector delivery.

**Methods:**

A combination of the following modifications scored best regarding the preservation of closed, native-like Env trimer conformation and antigenicity when using a panel of selected broadly neutralizing (bnAb) and non-neutralizing (nnAb) monoclonal antibodies for flow cytometry: i) replacing the natural cleavage site with a native flexible linker and introducing a single amino acid substitution to prevent CD4 binding (*), ii) fusing a heterologous VSV-G-derived transmembrane moiety to the gp140 C-terminus, and iii) deleting six residues proximal to the membrane.

**Results:**

When delivering membrane-tethered sC23v4 KIKO* and ConCv5 KIKO* via DNA, VSV-GP, and NYVAC vectors, the two native-like Env trimers provide differential antigenicity profiles. Whereas such patterns were largely consistent among the different vectors for either Env trimer, the membrane-tethered ConCv5 KIKO* trimer adopted a more closed and native-like structure than sC23v4 KIKO*. In immunized mice, VSV-GP and NYVAC vectors expressing the membrane-tethered ConCv5 KIKO* administered in prime/boost combination were the most effective regimens for the priming of Env-specific CD4 T cells among all tested combinations. The subsequent booster administration of trimeric ConCv5 KIKO* Env protein preserved the T cell activation levels between groups. The evaluation of the HIV-1-specific humoral responses induced in the different immunization groups after protein boosts showed that the various prime/boost protocols elicited broad and potent antibody responses, preferentially of a Th1-associated IgG2a subclass, and that the obtained antibody levels remained high at the memory phase.

**Discussion:**

In summary, we provide a feasible strategy to display multiple copies of native-like Env trimers on the cell surface, which translates into efficient priming of sustained CD4^+^ T cell responses after vector delivery as well as broad, potent, and sustained antibody responses following booster immunizations with the homologous, prefusion-stabilized, close-to-native ConCv5 KIKO* gp140 Env trimer.

## 1 Introduction

Since the identification of the human immunodeficiency virus type 1 (HIV-1) as the causative agent of acquired immunodeficiency syndrome (AIDS) in 1983 (1), many attempts were undertaken to eradicate HIV-1. The latest estimates indicate that 39 million people are living with HIV/AIDS worldwide, 1.3 million people are newly infected with HIV, and 630,000 persons died of AIDS-related illnesses in 2022, with sub-Saharan Africa remaining the region most severely impacted by the HIV/AIDS pandemic (https://www.unaids.org/en). Despite the success of the life-saving combined antiretroviral therapy (cART) in controlling the infection (2) and its availability to a growing number of individuals worldwide, the establishment of latent HIV-1 reservoirs makes the total eradication of the virus in cART-treated infected people extremely difficult (3, 4). Accordingly, a safe and effective vaccine able to prevent the acquisition of HIV-1 or at least the progression of the disease is necessary but still lacking.

To date, only the HIV-1 phase III Thai RV144 clinical trial has shown some efficacy (31.2%) against HIV-1 acquisition. This prophylactic trial used a prime/boost immunization regimen including the canarypox vector ALVAC and alum-adjuvanted AIDSVAX B/E, a bivalent monomeric HIV-1 glycoprotein 120 (gp120) (5). This trial provided relevant information on the immunogenicity and efficacy of the vaccination protocol, and, at present, more knowledge is obtained from the analysis of the vaccine-induced immune correlates of protection in non-human primates (NHPs) and especially in the efficacy trials that have been conducted in humans (6–8). On the basis of these analyses, the immune correlates that have been postulated to provide protection comprise a complex combination of individual immune effectors (9) such as i) non-neutralizing binding antibodies targeting the variable loops 1 and 2 (V1/V2) of the HIV-1 Env protein (10), particularly when the antibody isotype was IgG3 (11); ii) Env-specific polyfunctional antibodies, with antibodies that can mediate antibody-dependent cellular cytotoxicity (ADCC) (5), but only in the absence of serum IgA responses (12); and iii) T cell responses that can also contribute to protection (13). Furthermore, there is broad consent that protective HIV-1 vaccines will also have to induce broadly neutralizing antibodies (14). Therefore, a vaccine candidate or a specific vaccination regimen should elicit polyfunctional responses from both arms of the immune system but with optimal immune features.

Parameters that affect, among others, the outcome of immune responses include the antigen design, the delivery system, the administration route, and the number and spacing of immunizations. In addition, the combination of different vector systems including adjuvanted protein formulations in heterologous prime/boost immunization regimens has been reported to elicit more potent immune responses than homologous vectors (15). In this context, major efforts have been devoted to establishing the best-in-class combination of both prophylactic and therapeutic vaccine candidates in preclinical and clinical trials (16–19). The most advanced phase 2b/3 prophylactic clinical trials included the combination of the poxvirus clade C canarypox ALVAC (Env/Gag/Pro)/MF59-adjuvanted monomeric gp120 vaccine (HVTN 702 study; https://clinicaltrials.gov/ct2/show/NCT02968849), the trivalent adenovirus 26 vector expressing mosaic Env/Gag/Pol + clade C gp140 envelope protein with alum in sub-Saharan Africa (HVTN 705 HPX2008; https://clinicaltrials.gov/ct2/show/NCT03060629), and the tetravalent adenovirus 26 vector expressing mosaic Env/Gag/Pol + bivalent mosaic/clade C gp140 envelope protein with alum in the Americas and Europe (HVTN 706 HPX3002; https://clinicaltrials.gov/ct2/show/NCT03964415). Thus far, vaccinations in the HVTN 702 trial were discontinued early due to non-efficacy in providing protection against HIV-1 infection. Similarly, the adenovirus 26 HVTN 705 and 706 studies (Imbokodo and Mosaico) were also stopped due to a lack of protective efficacy. Considering the limited capacity of the current vaccination regimens to control HIV-1 infection, protocols and immunogens that can enhance and improve the HIV-1-specific B and T cell immune responses are needed.

The encouraging results of the control of the Ebola epidemic using a vesicular stomatitis virus (VSV)-based recombinant vector expressing the glycoprotein (GP) of the Zaire strain of Ebola virus (20) has increased the interest in this virus as a vector delivery system. We recently established a chimeric VSV vector by substituting the glycoprotein G of VSV for the glycoprotein GP of the lymphocytic choriomeningitis virus (VSV-GP) as an HIV-1 vaccine vector. VSV-GP expressing different HIV-1 Env variants readily elicited HIV-1-specific antibodies in rabbits and mice after homologous immunizations (21). Further, a combination of recombinant DNA, VSV-GP, or NYVAC vectors expressing membrane-tethered clade C 96ZM651 gp145 Env protein can induce higher and/or more balanced HIV-1-specific cellular and humoral immune responses in mice compared to the homologous vector combination (22). Prime/boost combinations of NYVAC expressing clade C GagPolNef and gp140 Env or a DNA vaccine encoding matched Gag, PolNef, and Env (DNA PT123) and recombinant Env protein in adjuvant induced in both NHPs and human clinical trials broad HIV-1-specific B and T cell responses, as well as neutralizing antibodies mainly directed against tier 1 HIV-1 viruses (23–26). An African-led phase 2b efficacy trial (PrEPVacc) testing our DNA-PT123 vaccine in combination with AIDSVAX B/E Env protein is still underway (https://clinicaltrials.gov/ct2/show/NCT04066881).

As a continuation of our previous studies on HIV-1 vaccines, we aimed herein to optimize two advanced prefusion-stabilized clade C trimers, which were developed under the Horizon 2020 European HIV Vaccine Alliance (EHVA), for membrane display and delivery from different non-viral (DNA) and viral (VSV-GP and NYVAC) vectors. Furthermore, we set out to determine the impact of vector-mediated delivery of such membrane-tethered prefusion-stabilized Env trimers on the priming of T and B cell responses and the quality and magnitude of such responses following two booster immunizations with the homologous soluble, prefusion-stabilized Env protein trimer in mice.

## 2 Materials and methods

### 2.1 Env construct designs

The coding sequences of the two novel prefusion-stabilized clade C Env trimers, sC23v4 KIKO and ConCv5 KIKO, used in this study were derived from a patient isolate (GenBank accession: AY255826) and a newly calculated clade C consensus sequence, respectively (27). sC23v4 KIKO and ConCv5 KIKO constructs contained an identical set of modifications comprising i) the replacement of the endogenous signal peptide by the tissue plasminogen activator (tPA) signal peptide; two sets of modifications to stabilize Env trimers in a prefusion state including ii) either a set of SOSIP mutations [A501C-T605C, I559P and the natural REKR furin cleavage site replaced by R6 (28–30)] or iii) a set of NFL mutations replacing the furin cleavage site by a native flexible linker (NFL; (G_4_S)_2_) in combination with the I559P substitution (31); iv) a set of point mutations following design principles described by Guenaga and colleagues (32) and the combination of I535M and Q543N substitutions (33) to further support prefusion trimer stabilization; v) the combination of H66R and T316W substitutions to impede transition to the CD4i state; vi) a second disulfide bond A73C-A561C between gp120 and gp41 (34); and vii) a G473T point mutation in order to abolish CD4 receptor engagement (35). In addition, N-linked glycans required for binding of bnAb 2G12 were introduced, if not naturally present (36), and N-linked glycans at positions N276 and N460/N463 around the CD4 binding site were deleted by substitution of asparagine with glutamine (37), collectively referred to as “KIKO 473T”. gp140 constructs comprising modifications i), ii), and iv)–vii) are referred to as “SOSIP”, while constructs comprising modifications i) and iii)–vii) are referred to as “NFL”.

For membrane display, such sC23v4 KIKO and ConCv5 KIKO derived prefusion-stabilized Env trimers were tethered to the membrane either i) by truncating Env at position 712 within the gp41 cytoplasmic tail yielding gp145 Env or ii) by fusing the VSV-G-derived transmembrane domain and cytoplasmic tail (aa 462-511) to gp140 Env truncated at position 678 (referred to as gp140:G), in principle as described earlier (20). To further optimize the display of the prefusion-stabilized Env, we used TMpred (38) or better prediction of the membrane-spanning domain within VSV-G. As a result, and in order to achieve optimal display of the native prefusion-stabilized HIV Env on the membrane, six N-terminal residues of the VSV-G-derived transmembrane domain (KSSIAS, aa 462-467) were replaced by isoleucine and fused to Env at position 683 (referred to as gp140:GΔ6).

For reference, the coding sequence of the clade C Env 96ZM651 (GenBank accession: AF286224) was modified by replacement of the furin cleavage site REKR by REKS for inhibition of cleavage and the sequence was truncated after position 712 for membrane tethering (gp145 96ZM651).

For the expression of soluble gp140 trimers, the coding sequences after position 664 were truncated, and an Avi-His (6) tag was fused to support the purification of secreted Env trimers.

All coding sequences were RNA- and codon-optimized for human codon usage, and all numbering of Env aa positions given was relative to the HXB2 sequence.

The above set of membrane-tethered stabilized sC23v4 KIKO and ConCv5 KIKO derivatives was subcloned into pcDNA5-FRT/TO for initial downselection in flow cytometry (see Section 2.4).

### 2.2 Cells

African green monkey kidney cells (BSC-40; ATCC catalog no. CRL-2761; ATCC, Manassas, VA, USA), human HeLa cells (ATCC, CCL-2), baby hamster kidney fibroblasts (BHK-21; ATCC, CCL-10), and primary chicken embryo fibroblast (CEF) cells were grown in Dulbecco’s Modified Eagle’s Medium (DMEM; Sigma-Aldrich, St. Louis, MO, USA) supplemented with 0.1 mM non-essential amino acids (Sigma-Aldrich), 2 mM l-glutamine (Merck, Whitehouse Station, NJ, USA), 100 U/mL penicillin/100 μg/mL streptomycin (Sigma-Aldrich), 0.5 μg/mL amphotericin B (Fungizone; Gibco-Life Technologies, Waltham, MA, USA), and 10% heat-inactivated newborn calf serum (NCS; Sigma-Aldrich) for BSC-40 and HeLa cells or fetal calf serum (FCS; Sigma-Aldrich) for BHK-21 and CEF cells. HEK293T cells (ATCC, CRL-3216) were grown in DMEM (high glucose, pyruvate; Gibco-Life Technologies) supplemented with 100 U/mL penicillin/100 µg/mL streptomycin (PAN Biotech, Aidenbach, Germany) and 10% FCS (Sigma-Aldrich). The above cell lines were maintained in a humidified 5% CO_2_ atmosphere at 37°C. FreeStyle™ 293-F cells were grown in FreeStyle™ 293 Expression Medium (Gibco-Life Technologies) supplemented with 100 U/mL penicillin/100 µg/mL streptomycin (PAN Biotech) and maintained in an 8% CO_2_ atmosphere at 37°C in an orbital shaker at 90 rpm.

### 2.3 Generation of non-viral and viral vectors

DNA vaccines were generated by subcloning gp140:GΔ6 sC23v4 KIKO 473T NFL or gp140:GΔ6 ConCv5 KIKO 473T NFL and, for control, gp145-96ZM651 into pVRC8400 (shortly termed DNA-sC23v4 KIKO*, DNA-ConCv5 KIKO*, and DNA-gp145-96ZM651, respectively). Plasmids used for immunization were produced in *Escherichia coli* DH5α and purified using the EndoFree Plasmid Giga Kit (Qiagen, Hilden, Germany) following the manufacturer’s instructions. The resulting purified plasmids were resuspended in phosphate-buffered saline (PBS) at 2 mg/mL, and supercoil-content, identity, and absence of endotoxin were quality-controlled.

VSV-GP-based viruses used in this work included VSV-GP, VSV-GP-gp145-96ZM651 (shortly termed VSV-GP-gp145), VSV-GP-gp140:GΔ6 sC23v4 KIKO 473T NFL (shortly termed VSV-GP-sC23v4 KIKO*), or VSV-GP-gp140:GΔ6 ConCv5 KIKO 473T NFL (shortly termed VSV-GP-ConCv5 KIKO*). VSV-GP (39) and VSV-GP-gp145 (22) have been previously described. VSV-GP-sC23v4 KIKO* and VSV-GP-ConCv5 KIKO* were generated by exchanging the luciferase gene in VSV-GP-Luc (40) via *Xho*I/*Nhe*I sites with the Env coding insert obtained by PCR from the corresponding pVRC8400-based plasmids. The resulting viruses, VSV-GP-sC23v4 KIKO* and VSV-GP-ConCv5 KIKO*, were recovered via reverse genetics using a helper virus-free protocol. Viruses were plaque-purified twice on BHK-21 cells and amplified on 293-F cells. Virus supernatants were harvested, underwent low-speed overnight centrifugation through a 20% sucrose cushion, and resuspended in PBS. Viruses were stored at −80°C in aliquots and titrated on BHK-21 cells via TCID_50_ assay. *In vitro* and *in vivo* characterization of these VSV-GP-based recombinant viruses will be described in detail elsewhere (manuscript in preparation).

The poxviruses used in this work included the genetically attenuated vaccinia virus (VACV)-based vector NYVAC-WT (kindly provided by Sanofi-Pasteur) and the recombinant viruses NYVAC-GPN, expressing Gag(96ZM651)-Pol-Nef(97CN54) as virus-like particles (41), NYVAC-gp145, expressing a membrane-bound trimeric gp145 (96ZM651) (22), and NYVAC-sC23v4 KIKO* and NYVAC-ConCv5 KIKO*, expressing the novel HIV-1 clade C membrane-bound trimeric gp140:GΔ6 sC23v4 KIKO 473 NFL and gp140:GΔ6 ConCv5 KIKO 473T NFL Env, respectively. PCR fragments encoding the respective Env KIKO* inserts were generated from the corresponding pVRC8400 plasmids and inserted via *Xho*I and *Pac*I into the plasmid transfer vector pLZAW1. The resulting plasmids were used for the insertion of the respective transgenes into the viral thymidine kinase (TK) locus (*J2R* gene) of the parental NYVAC-WT by homologous recombination to generate NYVAC-sC23v4 KIKO* and NYVAC-ConCv5 KIKO*. *In vitro* recombination, serial plaque purification, generation of virus stocks, and determination of virus titers were performed essentially as described earlier (22, 42). Further analysis of NYVAC-sC23v4 KIKO* and NYVAC-ConCv5 KIKO* recombinant viruses comprised the following: i) PCR-based quality control checking for transgene identity and virus homogeneity, ii) assessment of virus growth kinetics, iii) time-course analysis of transgene expression, and iv) virion localization of the transgene (**Supplementary Methods 1.1–1.5** and **Supplementary Figure 1**).

### 2.4 Characterization of Env variants on the surface of transiently transfected (DNA) or infected (VSV-GP or NYVAC) cells by flow cytometry

For the characterization of membrane-bound Env constructs, HEK293T cells were transiently transfected in triplicate either with the pcDNA5-FRT/TO plasmids encoding the corresponding *Env* genes for initial downselection or with the pVRC8400-based vaccines DNA-sC23v4 KIKO*, DNA-ConCv5 KIKO*, and DNA-gp145-96ZM651 using linear polyethylenimine (PEI 25K™; Polysciences, Warrington, PA, USA) as transfection reagent. Briefly, 3 × 10^5^ cells/well were seeded in a 96-well plate 16–18 h prior to transfection in 100 µL culture medium. On the day of transfection, the culture medium was replaced with 30 µL DMEM without supplements; 30 µL of transfection mix containing 240 ng of plasmid DNA and PEI at a fivefold excess relative to the DNA amount in DMEM was added. For SOSIP constructs, an Env to Furin ratio of 3:1 was used, and for uncleaved Envs (NFLs and 96ZM651 gp145), furin DNA was replaced by vector without any insert. After 5–6 h, the transfection mix was replaced by culture medium, and transfected cells were analyzed 2 days post-transfection by flow cytometry. For this, cells were washed with PBS and resuspended in FACS buffer (PBS 1×, 1% heat-inactivated FCS, 2 mM ethylenediaminetetraacetic acid (EDTA), and 0.1% NaN_3_). Primary anti-Env antibodies were 1:4 serially diluted in FACS buffer in seven steps starting at 10 µg/mL and incubated for 1 h at 4°C. Plates were then washed three times with FACS buffer and the secondary goat anti-human IgG (H+L)-Alexa647 antibody (Jackson ImmunoResearch, Cambridgeshire, UK) was added in a 1:100 dilution in FACS buffer and incubated for 30 min at 4°C. Following extensive washing with FACS buffer, cells were analyzed in an Attune NxT Flow Cytometer (Thermo Fisher Scientific, Waltham, MA, USA). Binding is given as mean fluorescence intensity (MFI; Alexa-647, RL1 channel) of cells gated as positive based on control-transfected cells (Env-specific signal). Antibody binding was then calculated as the area under the titration curve (AUC) of the MFI over this dilution range.

For the characterization of Env trimers displayed on the surface of VSV-GP-infected cells, HEK293T cells were infected at 0.1 TCID_50_/cell with the respective VSV-GP variant. VSV-GP without an insert was used as the negative control. At 24 h after infection, cells were resuspended and washed once with FACS buffer (PBS 1×, 1% FCS, and 0.05% NaN_3_), and ~2 × 10^5^ cells per staining were used in duplicate samples. To identify infected cells, the LCMV-GP-specific Wen4 hybridoma supernatant was used. Cells were fixed for 15 min with 1.5% formaldehyde at room temperature (RT) and washed once with FACS buffer. Cells were stained with primary anti-Env (10 µg/mL) or anti-GP (Wen4, in-house produced hybridoma supernatant 1:10 diluted) antibodies for 1 h at 4°C, washed once, and stained with donkey anti-human IgG (H+L)-Cy5 (1:200; Jackson ImmunoResearch) or goat anti-mouse IgG (H+L)-APC (1:100; Invitrogen, Carlsbad, CA, USA) secondary antibodies for 30 min at 4°C. Cells were then washed twice with FACS buffer, fixed with 1.5% formaldehyde, and quantified using FACS CantoII and Diva software. The Env-specific signal is defined as the geometric mean fluorescence (Cy5 signal) for each antibody normalized to the signal of VSV-GP empty-infected cells and was normalized for infection via the LCMV-GP staining (APC signal).

Finally, for the characterization of Env trimers on the surface of non-permeabilized NYVAC-infected cells, HeLa cells were infected at 3 pfu/cell with NYVAC-GPN (negative control), NYVAC-gp145, NYVAC-sC23v4 KIKO*, or NYVAC-ConCv5 KIKO* viruses. At 16 h post-infection, cells were washed twice with PBS (no magnesium/calcium; Gibco-Life Technologies), harvested with PBS 1× and 2 mM EDTA, washed with FACS buffer [PBS 1×, 2 mM EDTA, and 1% bovine serum albumin (BSA)], and centrifuged at 1,500 rpm for 5 min. Cells were then incubated with live/dead fixable red dye (1:200; Invitrogen) in the dark for 30 min at 4°C, washed twice with FACS buffer, and blocked with PBS 1× and 3% BSA for 30 min at 4°C. Primary human IgG anti-Env broadly neutralizing antibodies (bnAbs) or non-neutralizing antibodies (nnAbs) at 10 μg/mL in 50 μL FACS buffer were incubated with 10^6^ cells in the dark at 4°C for 30 min. After, cells were washed twice with FACS buffer and incubated with secondary F(ab′)2-goat anti-human IgG (H+L)-PE antibody (1:100; Beckman Coulter, Brea, CA, USA) in 50 μL FACS buffer in the dark at 4°C for 30 min. Next, cells were washed twice with FACS buffer and fixed with 0.5% formaldehyde. Finally, samples were acquired in an FC500 Laser flow cytometer (Beckman Coulter), and data analysis was performed using FlowJo software (Version 10.4.2; Tree Star, Ashland, OR, USA). The Env-specific signal is given as the geometric MFI of cells in the “live cells” gate multiplied by the percentage of cells gated as positive based on control cells infected with NYVAC-GPN (negative control).

For the above analyses, a panel of human bnAbs targeting V1/V2 quaternary N-glycans (PGT145 and PG16), V3 N-glycans (10-1074 and PGT121), V3 loop (447-52D), CD4 binding site (VRC01), gp120/gp41 interface (PGT151, 3BC176, and 35O22), outer domain (OD) glycans (2G12), or membrane proximal external region (MPER) (10E8) epitopes located on the Env trimer was used. The following nnAbs were also included: 17b (targeting CD4i), 39F (targeting V3 N-glycans), and F105 (targeting CD4 binding site) (see below).

### 2.5 Monoclonal antibodies

Monoclonal antibodies (mAbs) 5F3 (AB001) and 2G12 (AB002) were purchased from Polymun Scientific Immunbiologische Forschung GmbH (Klosterneuburg, Austria). Antibodies and plasmids encoding PGT145 and PGT151 antibodies were kindly provided by Dennis Burton (The Scripps Research Institute, La Jolla, CA, USA). Antibodies and plasmids encoding 10-1074 and 3BC176 antibodies were kindly provided by Michel Nussenzweig (The Rockefeller University, New York, NY, USA). The following reagents were obtained from the NIH HIV Reagent Program, Division of AIDS, NIAID, NIH: Human anti-HIV-1 gp120 V3 recombinant antibody (clone 39F); anti-HIV-1 gp120 mAb PG16 (ARP-12150); anti-HIV-1 gp120 mAb PGT121 (ARP-12343) was contributed by International AIDS Vaccine Initiative; anti-HIV-1 gp120 mAb VRC01 heavy chain (ARP-12035) and light-chain (ARP-12036) expression vectors were contributed by Dr. John Mascola; anti-HIV-1 gp120 mAb F105 (ARP-857) was contributed by Dr. Marshall Posner and Dr. Lisa Cavacini; HIV-1 447-52D mAb heavy chain (ARP-13617) and light-chain (ARP-13618) expression vectors were contributed by Dr. Susan Zolla-Pazner; anti-HIV-1 gp120 mAb clone 17b (ARP-4091) and anti-HIV-1 gp120-V3 mAb (ARP-11437) were contributed by Dr. James E. Robinson; anti-HIV MPER mAb 10E8 heavy chain (ARP-12290) and light-chain (ARP-12291) expression vectors, as well as anti-gp120/gp41 interface mAb 35O22 heavy chain (ARP-12584) and light-chain (ARP-12585) expression vectors, were contributed by Dr. Mark Connors. In-house production of human anti-Env mAbs was performed in FreeStyle™ 293-F cells (Gibco-Life Technologies) or Expi293F™ cells (Thermo Fisher Scientific) as described earlier (27).

### 2.6 Recombinant Env proteins

The panel of Env proteins used in this study for serological readout comprised prefusion-stabilized, closed conformation trimers from clades A (BG505 SOSIP), B (AMC008 SOSIPv4.2 and AMC011 SOSIPv4.2), and C (16055 SOSIP, sC23v4 KIKO SOSIP 473T (shortly termed sC23v4 KIKO*), ConCv5 KIKO SOSIP (shortly ConCv5 KIKO), and ConCv5 KIKO 473T SOSIP [shortly termed ConCv5 KIKO*)], as well as two open trimers from clade C (ConCv1 NFL KIKO and 96ZM651 REKS.664) and one clade C gp120 monomer (ConC KIKO) as described earlier (27). Prefusion-stabilized, closed conformation ConCv5 KIKO* was also used as an antigen in immunization experiments. Envelope proteins were expressed in transiently transfected FreeStyle™ 293-F cells and purified by immobilized metal affinity chromatography (IMAC) followed by size exclusion chromatography (SEC) as described previously (27). Protein-containing fractions were analyzed for trimer content by Blue Native PAGE (SERVAGel™ N 4-16; SERVA, Heidelberg, Germany). For analytical purposes, 10 µg of the final pool was analyzed by SEC, and 2 µg was loaded on a Blue Native PAGE as well as on reducing and non-reducing sodium dodecyl sulfate–polyacrylamide gel electrophoresis (SDS-PAGE) (SERVAGel™ TG PRiME™ 8%–16%; SERVA).

### 2.7 Peptides

The HIV-1 consensus subtype C gp160 peptide pool was obtained from the AIDS Reagent Program, Division of AIDS, NIAID, NIH. This pool covered the HIV-1 gp160 protein from consensus clade C included in DNA-ConCv5 KIKO*, VSV-GP-ConCv5 KIKO*, and NYVAC-ConCv5 KIKO* vectors as consecutive 15-mers overlapping by 11 amino acids.

### 2.8 Ethics statement

Experimental protocols for the *in vivo* assays in mice were authorized by the Ethical Committee of Animal Experimentation of Centro Nacional de Biotecnología (CEEA-CNB, Madrid, Spain) in agreement with International EU Guidelines 2010/63/UE on protection of animals for scientific purposes, Spanish National Law 32/2007 on animal welfare, exploitation, transport, and sacrifice, and Spanish National Royal Decree RD 53/2013 (permit number PROEX 281/16).

### 2.9 Mouse immunization

Groups of female 6–8-week-old BALB/c mice purchased from Envigo (Gannat, France) (n = 10) were immunized with 1 × 10^7^ TCID_50_ of VSV-GP-ConCv5 KIKO* (V), 1 × 10^7^ pfu of NYVAC-ConCv5 KIKO* (N), 50 μg of DNA-ConCv5 KIKO* (D), or 20 μg of prefusion-stabilized, close-to-native ConCv5 KIKO* Env trimer (P) + 50 μg of MPLA (Polymun Scientific Immunbiologische Forschung GmbH) as adjuvant (Adj.) by bilateral intramuscular (i.m.) route (groups 1–5). MPLA was selected as adjuvant based on i) robustness of antibody responses observed in previous preclinical assessments (22, 27, 43–45), ii) proven safety in humans in combination with other HIV-1 Env proteins (https://clinicaltrials.gov/study/NCT03961438, https://clinicaltrials.gov/study/NCT05208125), and iii) availability for testing our ConCv5 KIKO* candidate in future phase 1 clinical trials. Four weeks later, animals were immunized with NYVAC, VSV-GP, or DNA vectors or with ConCv5 KIKO* Env protein as in the priming in homologous (groups 2, 3, 4, and 5) or heterologous (group 1) combinations. Mice primed with 1 × 10^7^ TCID_50_ of VSV-GP-WT and boosted with 1 × 10^7^ pfu of NYVAC-WT were used as control group (group 6). At 10 days post-boost, half of the animals of each group (n = 5) were sacrificed, the spleens and inguinal plus popliteal draining lymph nodes (DLNs) for each group were pooled and processed by meshing through a cell strainer for Intracellular Cytokine Staining (ICS) assay, and sera from individual mice were collected for enzyme-linked immunosorbent assay (ELISA) to determine both cellular and humoral acute immune responses against HIV-1 ConCv5 KIKO* protein. At weeks 8 and 12, animals from groups 1–5 (n = 5) were boosted with 20 μg of ConCv5 KIKO* Env trimer + 50 μg of MPLA as adjuvant by i.m. route. Animals from group 6 (n = 5) only received MPLA at weeks 8 and 12. At 90 days post-second protein boost, the remaining five animals of each group were sacrificed, the spleens and DLNs were processed for ICS assay, and sera were collected for ELISA to analyze both cellular and humoral memory immune responses against HIV-1 ConCv5 KIKO* Env trimer. At 10 days post-second protein boost, blood was collected by submandibular bleeding to evaluate HIV-1 ConCv5 KIKO*-specific acute humoral immune responses.

### 2.10 Analysis of the cellular immune responses by ICS assay

#### 2.10.1 Analysis of the HIV-1 Env consensus C-specific T cell responses

To evaluate the magnitude and phenotype of the HIV-1 Env consensus C-specific T cells, 2 × 10^6^ splenocytes (depleted of erythrocytes) were seeded in 96-well plates and stimulated *ex vivo* for 6 h in complete Roswell Park Memorial Institute (RPMI; Sigma-Aldrich) 1640 medium (2 mM l-glutamine, 100 U/mL penicillin/100 μg/mL streptomycin, 10 mM Hepes, and 0.01 mM β-mercaptoethanol) with 10% FCS, monensin 1× (Invitrogen), 1 μL/mL Golgiplug (BD Biosciences, San Jose, CA, USA), anti-CD107a-FITC (BD Biosciences), and 5 μg/mL of the HIV-1 gp160 (consensus C) peptide pool (AIDS Reagent Program, Division of AIDS, NIAID, NIH). Non-stimulated (RPMI) samples were used as controls. After the stimulation period, cells were washed, incubated with surface markers, permeabilized (Cytofix/Cytoperm kit; BD Biosciences), and stained intracellularly. The LIVE/DEAD Fixable Violet Dead Cell Stain Kit (Invitrogen) was used to exclude dead cells. For the analysis of T cells, the following fluorochrome-conjugated antibodies were used: CD3-PE-CF594, CD4-APC-Cy7, CD8-V500, CD49b-Alexa700, and B220-PerCP for phenotypic analyses and CD107a-FITC, IL-2-APC, TNF-α-PE, and IFN-γ-PE-Cy7 for functional analyses. All antibodies were from BD Biosciences.

#### 2.10.2 Analysis of the HIV-1 Env consensus C-specific T follicular helper cell responses

For the evaluation of the magnitude of the HIV-1 Env consensus C-specific T follicular helper (Tfh) cells, 2 × 10^6^ splenocytes (depleted of erythrocytes) or cells from the DLNs were seeded in 96-well plates and stimulated *ex vivo* for 6 h in complete RPMI 1640 medium with 10% FCS, monensin 1× (Invitrogen), 1 μL/mL Golgiplug (BD Biosciences), anti-CD154 (CD40L)-PE (BD Biosciences), and 5 μg/mL of HIV-1 gp160 (consensus C) peptide pool. Non-stimulated (RPMI) samples were used as controls. After the stimulation period, cells were washed, incubated with surface markers, permeabilized, and stained intracellularly. The LIVE/DEAD Fixable Violet Dead Cell Stain Kit (Invitrogen) was used to exclude dead cells. For the analysis of Tfh cell responses, the following fluorochrome-conjugated antibodies were used: CD4-Alexa700, CD8-V500, CXCR5-PE-CF594, PD1(CD279)-APC-efluor780, and B220-PerCP for phenotypic analyses and CD154 (CD40L)-PE, IFN-γ-PE-Cy7, and IL-4-Alexa488 for functional analyses. All antibodies were from BD Biosciences.

#### 2.10.3 Analysis of the HIV-1 ConCv5 KIKO*-specific germinal center B cell responses

To determine the magnitude of the HIV-1 ConCv5 KIKO*-specific germinal center (GC) B cell responses, 2 × 10^6^ splenocytes (erythrocyte-depleted) or cells from the DLNs were seeded in 96-well plates in complete RPMI medium with 10% FCS, pelleted, and stained with Fixable Viability Stain 520 (FVS 520; BD Biosciences). Fc receptors were then blocked with anti-CD16/CD32 antibody (BD Biosciences), and cells were incubated with biotinylated ConCv5 KIKO* protein (Biotin-XX Microscale Protein Labeling Kit; Invitrogen; 0.3 μg/10^6^ cells) in the dark at 4°C for 30 min. Next, cells were washed and incubated with the following fluorochrome-conjugated antibodies for surface markers: CD3-FITC, CD38-PerCP-Cy5.5, IgD-APC-H7, B220-PE-Cy7, GL7-Alexa647, IgG1-BV421, CD19-Alexa700, and IgM-PE-CF594 (all from BD Biosciences).

For the analysis of the above HIV-1 clade C Env-specific T and B cell responses by flow cytometry, a GALLIOS flow cytometer (Beckman Coulter) was used to acquire between 10^5^ and 5 × 10^5^ lymphocyte-gated events. The analysis of the data was performed using FlowJo software (Version 10.4.2; Tree Star). To analyze the polyfunctional profile of the Env-specific T cell responses, Boolean combinations of single functional gates were generated to quantify the frequency of each response based on all the possible combinations of cytokine expression. Background responses in the unstimulated controls (RPMI) were subtracted from those obtained in stimulated samples for each specific functional combination.

### 2.11 Analysis of the humoral immune responses by ELISA

#### 2.11.1 Measurement of serum reactivity toward the native homologous ConCv5 KIKO* trimer

Antibody binding to the homologous prefusion-stabilized, close-to-native ConCv5 KIKO* Env trimer in serum was determined by direct (46) or capture (27) ELISA. For direct ELISA, individual sera from immunized animals were threefold serially diluted in duplicates. Levels of Env-specific total IgG binding antibodies or IgG1, IgG2a, or IgG3 subclasses were determined as the last dilution of serum that yielded three times the mean optical density at 450 nm (OD_450_) of the control group (endpoint titer; total IgG binding antibodies) or as the OD_450_ at a serum dilution of 1:8,100 (10 days post-boost) or 1:24,300 (10 and 90 days post-second protein boost) (IgG subclass binding antibodies). Binding of serum antibodies was also determined for the properly oriented ConCv5 KIKO* Env trimer by capture ELISA using Ni-NTA HisSorb Plates (Qiagen) to capture the close-to-native prefusion-stabilized Env trimer via the His6-tag as previously described (27). Endpoint total IgG titers were determined for the individual sera in duplicates essentially as described above for the direct ELISA. The absence of Env reactivity in pre-immune sera toward the respective immunogen was confirmed for each animal.

#### 2.11.2 Determination of breadth of serum response

The breadth of serum reactivity was determined against a panel of eight Env variants via binding antibody multiplex assay (BAMA). The panel consisted of stabilized, closed trimers from clades A (BG505 SOSIP), B (AMC008 SOSIPv4.2 and AMC011 SOSIPv4.2), and C (16055 SOSIP and sC23v4 KIKO*), as well as two rather open trimers from clade C (ConCv1 KIKO NFL and 96ZM651 REKS.664) and one clade C gp120 monomer (ConC KIKO). MagPlexAvidin Microspheres (Luminex, Austin, TX, USA; 2,000 beads/test) were washed with dilution buffer [DB: PBS 1×, 1% BSA, 2 mM EDTA, 0.05% Tween-20, and 0.1% ProClin™ 300 (Sigma-Aldrich)] and coated (250 beads/µL) with 5 µg/mL of biotinylated anti-His6 IgY (Thermo Fisher Scientific) in DB at RT for 1 h. After washing with DB, the trimeric Envs were captured at 15 µg/mL and the monomeric gp120 at an equimolar amount overnight at 4°C. After washing with DB, beads were washed again with blocking buffer [BF: PBS 1×, 0.1% (w/v) I-Block™ (Invitrogen), and 1× Roti®Block (Carl Roth, Karlsruhe, Germany)] and blocked at RT for 1 h. Next, the different bead regions were pooled and distributed in a 96-well plate (Greiner, Kremsmünster, Austria), and serum samples were added at a 1:80 dilution in 50 µL of BF and incubated at RT for 2 h. Next, beads were washed with BF and DB, and a PE-conjugated donkey anti-mouse IgG (H+L) AffiniPure F(ab’)_2_ fragment was added at 1:200 dilution and incubated at RT for 1 h. Next, beads were washed and resuspended in DB, and measurement was carried out using a MAGPIX® device (Luminex). The response was defined as positive if the specific background corrected signal for an individual mouse was higher than three times the mean signal of the control group (group 6). Reactivities of the individual sera against the different envelopes were expressed as background-adjusted MFI minus background-adjusted blank (MFI*). To determine the breadth score, the responses against each Env were averaged per mouse and presented as median and range.

### 2.12 Data analysis and statistics

To evaluate the level of Env surface display, a t-test with Bonferroni correction was performed. For the analysis of ICS data, a previously described statistical method that adjusts the values of the unstimulated (RPMI) samples and calculates confidence intervals (CIs) and *p* values was used (47, 48). Only HIV-1-specific responses significantly higher than non-stimulated RPMI samples are represented. When indicated, the values shown are background-subtracted. For the analysis of ELISA data, a one-way ANOVA test followed by Tukey’s honestly significant difference (Tukey’s HSD) method was performed.

## 3 Results

### 3.1 Generation of membrane-bound HIV-1 gp140 Env trimers with broad bnAb recognition

**Figure 1:**
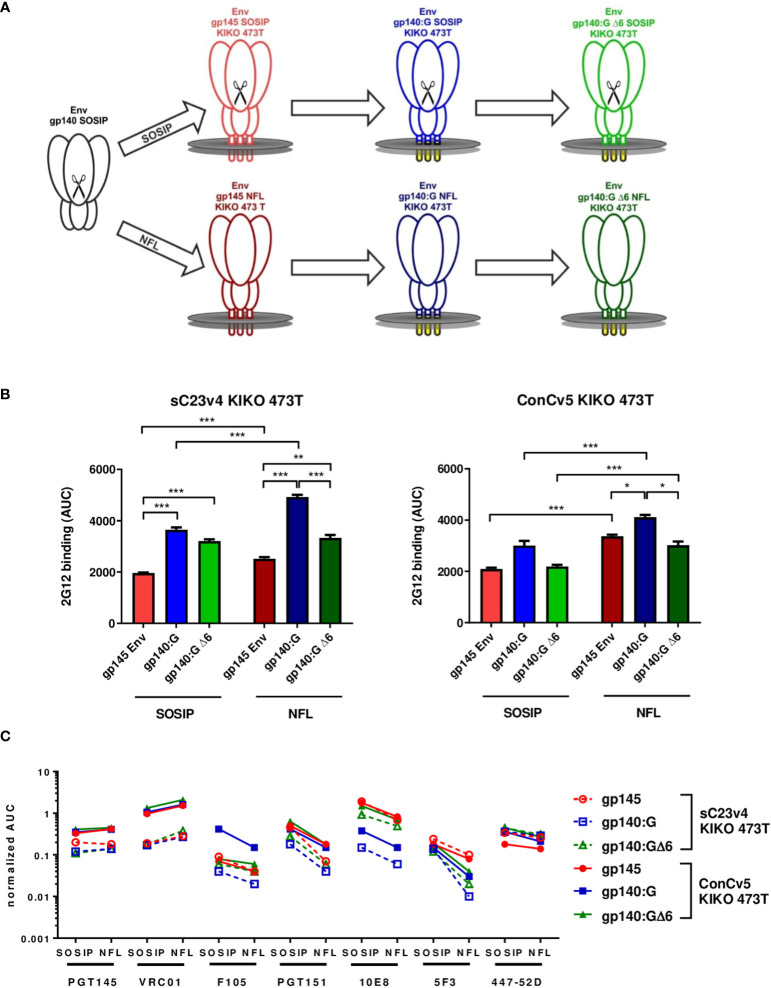
Strategies for membrane-tethering of native-like prefusion-stabilized Env trimers, levels of Env surface display, and Env antigenicity. **(A)** Soluble stabilized gp140 trimers (black) were sequentially optimized for display on membrane surfaces and generated as cleaved SOSIP (upper) and cleavage-independent NFL (lower) Env trimers. **(B)** Level of Env surface display. HEK293T cells were transfected in triplicate with the indicated pcDNA5-FRT/TO Env constructs encoding gp145 Envs (red), gp140:G (blue), and gp140:GΔ6 (green). Level of Env surface display is given as the area under the curve (AUC; mean ± SEM) following titration of mAb 2G12 on transfected cells and determination of mean fluorescence intensities (MFIs). *p < 0.05; **p < 0.01; ***p < 0.001 in t-test with Bonferroni correction. **(C)** MFI of the indicated broadly neutralizing antibodies (bnAbs) and non-neutralizing antibodies (nnAbs) measured by flow cytometry titration to the respective SOSIP and NFL Env variants normalized for the level of Env surface display.

The stabilized patient isolate-derived clade C Env variant sC23v4 KIKO and the clade C consensus Env variant ConCv5 KIKO that have originally been designed as soluble prefusion-stabilized, close-to-native gp140 SOSIP trimers (27) were re-designed for display on cell membranes. Three different membrane-bound variants were established and characterized (**Figure 1**): i) gp145 containing its autologous transmembrane domain and a cytoplasmic tail truncated (**Figure 1A**, upper panel, red), ii) a chimeric variant with the extracellular gp140 fused to the heterologous transmembrane domain of the VSV (VSV-TM) glycoprotein G (VSV-G) (Env gp140:G, blue) (21), and iii) a derivative thereof that was designed to retain binding of the MPER-directed bnAbs (gp140:GΔ6, green). In order to avoid the display of partially misfolded Env trimers (e.g., as a result of incomplete furin cleavage of SOSIP trimers after vector delivery), the two Envs were additionally remodeled to NFL-type constructs where the cleavage site was replaced by a flexible linker (**Figure 1A**, lower panel). Therefore, in summary, a total of six different variants (sC23v4 and ConCv5 derived SOSIP as well as NFL-type constructs encoding gp145, gp140:G KIKO 473T, and gp140:GΔ6 KIKO 473T) were subcloned into pcDNA5-FRT/TO for each of the two Envs (**Figure 1A**).

For the analysis of Env surface display, the sC23v4 KIKO 473T and ConCv5 KIKO 473T expression constructs were transfected into HEK293T cells and analyzed by flow cytometry (**Figures 1B, C**). The level of Env surface display was quantified using mAb 2G12 known to recognize a highly conserved high-mannose patch on gp120 (36). Except for ConCv5-gp140:GΔ6 KIKO 473T SOSIP and NFL types and ConCv5-gp140:G KIKO 473T SOSIP-type, the VSV-TM chimeric Env variants gp140:G and gp140:GΔ6 demonstrated significantly enhanced surface display compared to the gp145 (*p* < 0.001) (**Figure 1B**). In addition, higher surface expression of NFL-type Envs was observed compared to SOSIP-type Envs, which was statistically significant in all cases (*p* < 0.001) except for sC23v4-gp140:GΔ6 KIKO 473T (**Figure 1B**).

A native-like conformation of Env can be deduced from its reactivity to bnAbs targeting relevant sites of vulnerability (47) and its absence of reactivity to nnAbs (**Figure 1C**). ConCv5 KIKO 473T-derived membrane-tethered Envs showed superior or equivalent binding to their sC23v4 KIKO 473T counterparts (solid *vs.* dashed lines). This was most obvious for the bnAbs PGT145 (apex), VRC01 (CD4bs), PGT151 (interface), and 10E8 (MPER). NFL-type constructs overcoming the need for furin cotransfection scored at least equivalent to SOSIP-type constructs regarding PGT145 as well as VRC01 binding. As expected, binding of the cleavage-dependent antibody PGT151 was reduced in NFL-type constructs due to impaired accessibility of the fusion peptide (49). The nnAb F105, by trend, binds less well to the NFL-type trimers, suggesting reduced accessibility of nnAb-epitopes in the closed NFL conformations as compared to the SOSIP derivatives. Further evidence for at least partial preservation of the closed conformation following membrane-tethering of the Env trimers is provided by moderately, but not significantly, reduced binding to bnAb 447-52D. Binding of the MPER-directed bnAb 10E8 was significantly (*p* < 0.001) diminished in the gp140:G chimera but could be fully restored to gp145 levels in the gp140:GΔ6 chimera. Notably, binding to the MPER-directed nnAb 5F3 was reduced in NFL-type constructs with the reduction being especially pronounced for the two VSV-TM chimeric variants (**Figure 1C**).

Thus, the gp140:GΔ6 KIKO 473T NFL-type constructs (in the following sC23v4 KIKO* and ConCv5 KIKO*) exhibit superior properties and were selected for deeper *in vitro* and *in vivo* characterization analyses using a DNA vaccine vector (pVRC8400) or two viral vectors (VSV-GP and NYVAC) for antigen delivery.

### 3.2 Antigenicity of cell surface displayed sC23v4 KIKO* and ConCv5 KIKO* expressed from DNA vaccines and viral vectors

To determine whether the choice of vector for Env delivery has any significant impact on the conformation of our best-in-class membrane-tethered Env derivatives, the recognition profiles of sC23v4 KIKO* and ConCv5 KIKO* were next evaluated to a broader panel of human bnAbs and nnAbs. For that purpose, cells were transfected with a DNA vaccine vector (pVRC8400) or infected with VSV-GP (both HEK293T cells) or with NYVAC vectors (HeLa cells) expressing sC23v4 KIKO*, ConCv5 KIKO*, or a reference 96ZM651 gp145 protein by flow cytometry. The extended panel of bnAbs targets most of the vulnerable HIV-1 Env trimer epitopes reported (50).

**Figure 2:**
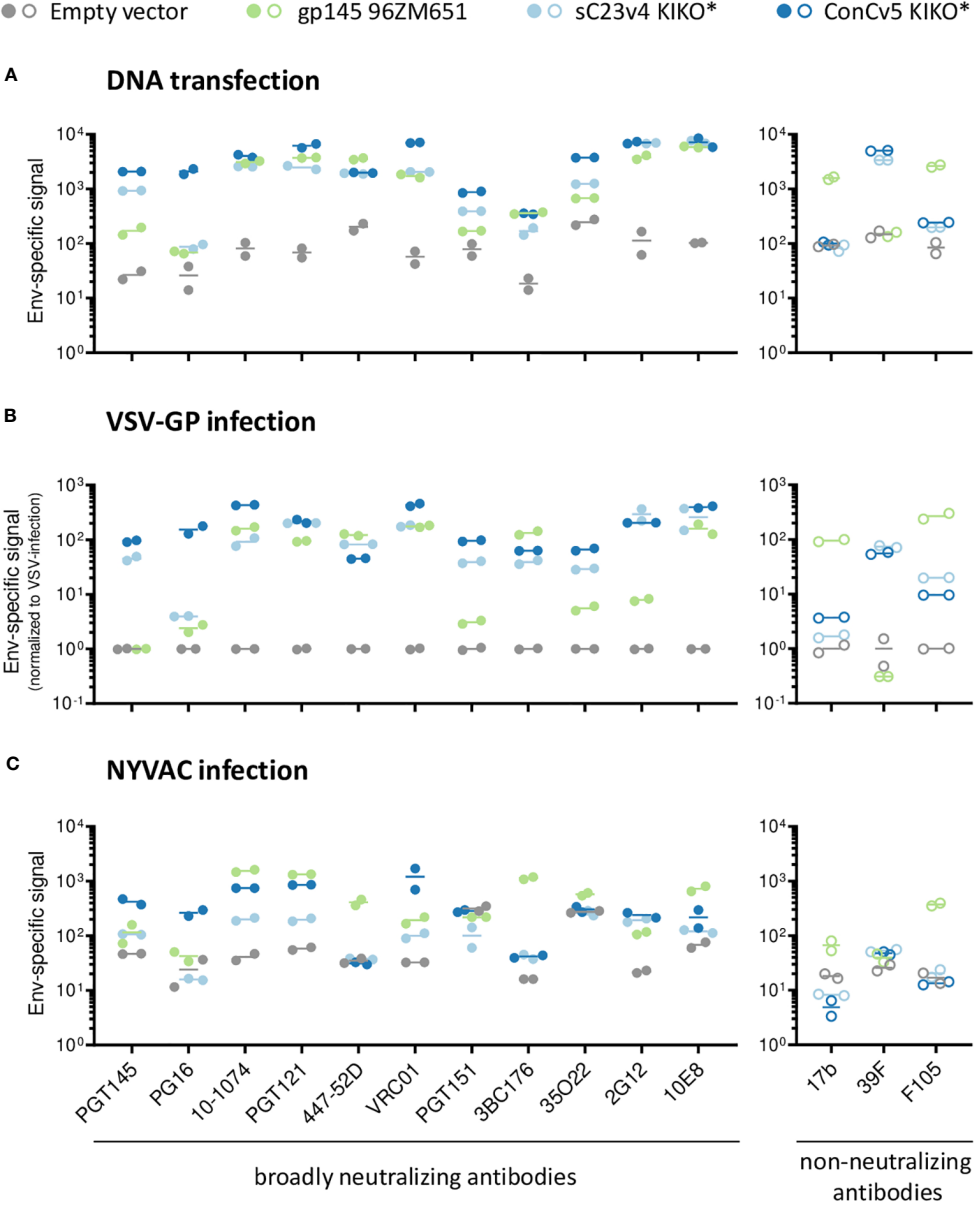
bnAb binding profile to membrane-displayed trimeric HIV-1 gp140:GΔ6 KIKO* proteins by flow cytometry. Antigenicity of membrane-bound Env displayed on the surface of DNA-transfected cells **(A)**, VSV-GP-infected cells **(B)**, both in HEK293T cells, or NYVAC-infected HeLa cells **(C)**. The respective empty vectors (gray) or vectors expressing 96ZM651 gp145 (green), membrane-tethered sC23v4 KIKO* (light blue), or ConCv5 KIKO* (dark blue) Env trimers were used for transient transfection or infection. Cells were stained with a set of primary human IgG anti-Env broadly neutralizing antibodies (bnAbs) (left panels, filled circles) and non-neutralizing antibodies (nnAbs) (right panels, open circles) and processed for flow cytometry. The broader panel of selected human bnAbs targets a V1/V2 quaternary structure element apex (PGT145 and PG16), V3 N-glycans (10-1074 and PGT121), V3 loop (447-52D), CD4bs (VRC01), gp120/gp41 interface (PGT151, 3BC176 and 35O22), OD-glycans (2G12), or MPER (10E8) epitopes on the Env trimer. The human nnAbs included in the analysis target CD4i (17b), V3 N-glycans (39F), and CD4 (F105). Results from two experiments (circles) and the mean (line) are shown.

A differential recognition profile was observed for sC23v4 KIKO* and ConCv5 KIKO* when subjected to the extended panel of mAbs (**Figure 2**). The most reactive cell surface displayed Env was ConCv5 KIKO*, with broader recognition in comparison to the sC23v4 KIKO* and our reference construct gp145 96ZM651. In particular, higher reactivity was observed to the bnAbs PGT145 and PG16 (V1/V2 quaternary epitopes), PGT121 (V3 N-glycans), VRC01 (CD4 binding site), and 35O22 (gp120/gp41 interface). For V1/V2 quaternary bnAbs, only the PGT145 was able to detect surface displayed sC23v4 KIKO* expressed from DNA (**Figure 2A**), VSV-GP (**Figure 2B**), or NYVAC (**Figure 2C**) vectors, but with lower affinity. bnAbs targeting V3 N-glycans, CD4 binding site, OD N-glycans, and MPER exhibited higher values in comparison with bnAbs targeting the quaternary structure and the gp120/gp41 interface (**Figure 2**). Regarding the accessibility of the V3 loop by the two tested V3-specific mAbs, bnAb 447-52D showed comparable binding to all three tested Env constructs when delivered via DNA and VSV-GP but was almost absent for sC23v4 KIKO* and ConCv5 KIKO* when delivered via NYVAC. V3-specific nnAb 39F readily detected membrane-tethered sC23v4 KIKO* and ConCv5 KIKO* when delivered via DNA and VSV-G, but Env signals for nnAb 39F remained at background levels when these two prefusion-stabilized trimers were delivered by NYVAC. Env signals for nnAb 39F also remained at background level for 96ZM651 gp145 regardless of the vector used, which can be explained by the absence of the sequence-specific 39F epitope in the V3 loop of 96ZM651 Env. Binding of 17b and F105, suggesting a more open structure, to sC23v4 KIKO* and ConCv5 KIKO* was clearly reduced and in some cases even close to empty vector as compared to 96ZM651 gp145, regardless of the applied delivery modality (**Figure 2**, right panels). Taken together, overall diminished reactivities of nnAbs 17b and F105 to sC23v4 KIKO* and ConCv5 KIKO* compared to 96ZM651gp145 suggest a more closed trimeric structure, which may help to focus B cell responses to vulnerable sites.

Despite minor differences regarding the antibody binding profiles, the type of vector system did not seem to have a significant effect on the overall antigenicity of the displayed envelopes. This similar behavior of sC23v4 KIKO* and ConCv5 KIKO* expressed from the three different vectors was also confirmed by correlation analyses yielding Spearman’s correlation coefficients of r = 0.97 (pcDNA5-FRT/TO vs. pVRC8400), r = 0.79 (VSV-GP *vs.* pVRC8400), r = 0.56 (NYVAC vs. pVRC8400), and r = 0.60 (NYVAC vs. VSV-GP), with *p* < 0.001 for all cases (**Supplementary Figure 2**).

In summary, these *in vitro* results showed that non-viral (DNA) and viral (VSV-GP and NYVAC) vectors express the gp140:GΔ6 KIKO 473T NFL trimers at the cell surface in a native-like conformation that is well recognized by a broad spectrum of human bnAbs, with superior antigenicity observed for the ConCv5 KIKO* compared to sC23v4 KIKO*. This differential antigenicity profile reflects the different conformations adopted by the prefusion-stabilized Env trimers following re-engineering for cell surface display and vector delivery, with ConCv5 KIKO* exhibiting a more closed structure than sC23v4 KIKO*. For this reason, ConCv5 KIKO* was selected for the *in vivo* analysis of the immunogenicity elicited by these non-viral and viral vectors in the mouse model.

### 3.3 Impact of G473T substitution on ConCv5 KIKO* trimer protein antigenicity

The antigenicity profile of ConCv5 KIKO (SOSIP) has been previously described in detail (27). In this study, we planned to use a modified variant, namely ConCv5 KIKO 473T (SOSIP) (shortly termed ConCv5 KIKO*), to boost vector-primed immune responses. The G473T substitution has been described to mitigate CD4 receptor binding, shown to prevent CD4-induced opening of otherwise stabilized Env trimers, and may also improve bioavailability following immunization (35).

Analytical size exclusion chromatography of the final ConCv5 KIKO* protein preparation revealed the presence of one major protein population (trimer) with only traces of impurities of other protein species (aggregate, dimer, and monomer) (**Supplementary Figure 3A**). Blue Native PAGE verified these findings (**Supplementary Figure 3B**); in addition, reducing and non-reducing SDS-PAGE confirmed complete cleavage of the gp140 trimer preparation used for vaccination (**Supplementary Figures 3C, D**). bnAb and nnAb binding characteristics of the purified ConCv5 KIKO* soluble trimer were assessed by Ni-NTA capture ELISA, and results were analyzed relative to the antigenicity of the parental prefusion-stabilized ConCv5 KIKO Env trimer (**Supplementary Methods 2** and **Supplementary Figure 3E**). Binding properties of trimer-specific PGT145, CD4bs VRC01, gp120/gp41 interface PGT151, and OD glycan-dependent 2G12 bnAbs were highly comparable between the two Env variants. bnAb 447-52D (targeting V3) and nnAbs 17b (detecting CD4i conformation) and F105 (targeting the CD4bs) did not bind the purified gp140 trimers (**Supplementary Figure 3E**).

Altogether, these results indicate that the G473T substitution in the CD4bs did not affect Env trimer formation or its antigenicity. Comparing the binding data of nnAbs obtained for purified ConCv5 KIKO* Env trimer to the data set obtained for the vector-delivered, membrane-tethered homolog (**Figure 2**, right panels), we conclude that the recombinantly expressed and carefully purified prefusion-stabilized Env trimer exhibits a more closed conformation as compared to its vector-delivered, membrane-displayed ConCv5 KIKO* counterpart.

### 3.4 HIV-1 Env-specific cellular and humoral immune responses induced in mice following ConCv5 KIKO* homologous and heterologous vector delivery

**Figure 3:**
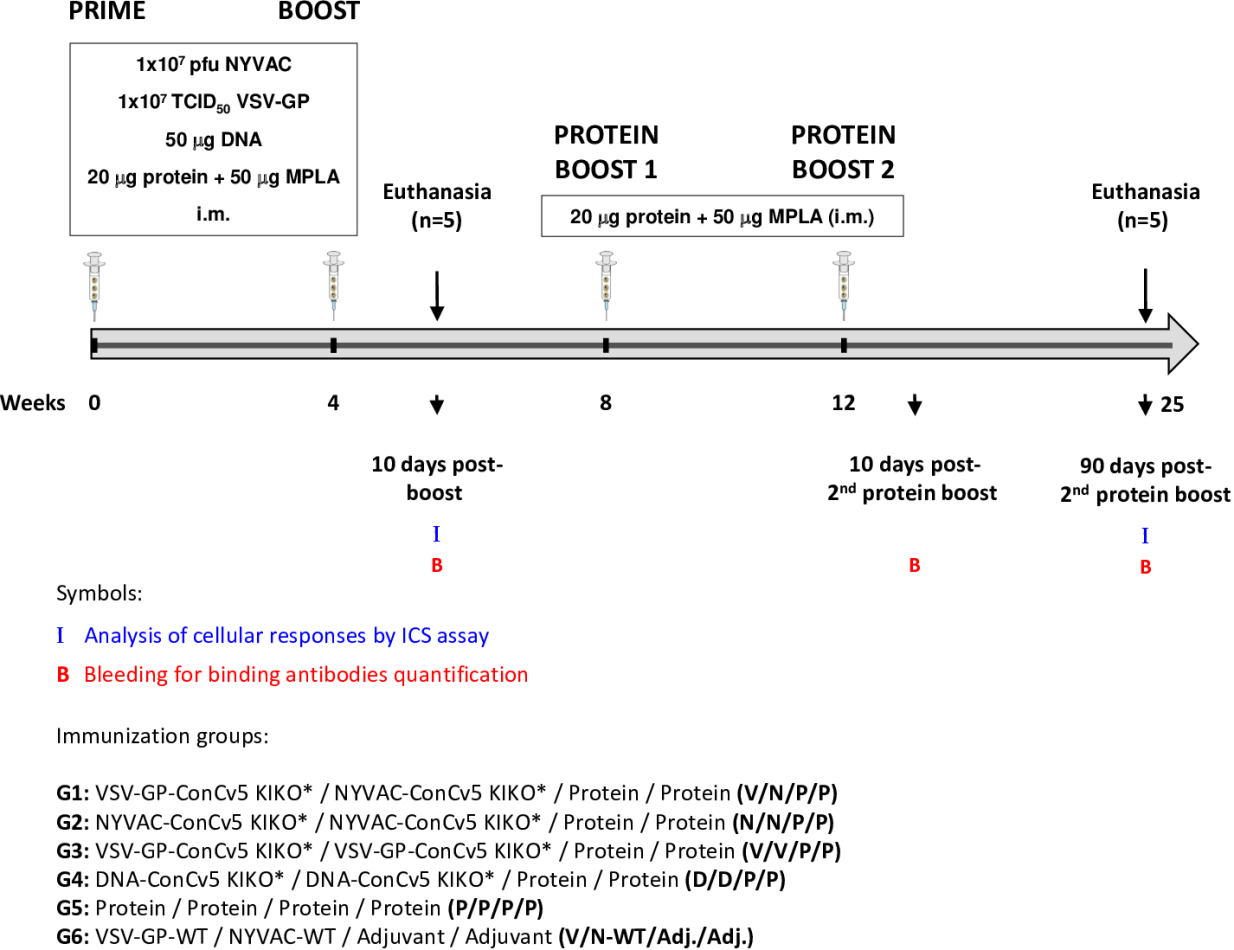
Immunization schedule for the evaluation of HIV-1 Env-specific immunogenicity in mice against ConCv5 KIKO* protein elicited by different homologous or heterologous prime/boost immunization regimens using non-viral and viral vectors, and recombinant protein. Female BALB/c mice (n = 10/group) received the indicated doses of VSV-GP-ConCv5 KIKO* (V), NYVAC-ConCv5 KIKO* (N), or DNA-ConCv5 KIKO* (D) vectors expressing trimeric or MPLA-adjuvanted trimeric ConCv5 KIKO* (SOSIP) recombinant protein (P) by i.m. route at weeks 0 and 4. At 10 days post-boost (w4 + 10 days), five animals of each group were sacrificed, and spleens and draining lymph nodes (DLNs) processed for Intracellular Cytokine Staining (ICS) assay and sera collected for ELISA to analyze cellular and humoral HIV-1 Env-specific acute immune responses, respectively, as described in Materials and Methods. At weeks 8 and 12, the remaining animals were boosted with 20 μg of ConCv5 KIKO* (SOSIP) prefusion-stabilized Env trimer + 50 μg of MPLA by i.m. route. Animals from group 6 received empty vectors and MPLA only adjuvant (Adj.). At 90 days post-second protein boost, animals were sacrificed, spleens and DLNs were processed for ICS assay, and sera were collected for ELISA to analyze cellular and humoral HIV-1 Env-specific memory immune responses. At 10 days post-second protein boost, blood was collected by submandibular bleeding to determine acute humoral immune responses. The different immunization groups are indicated.

An important requirement for Env-expressing vaccine candidate vectors is their capacity to potently activate HIV-1-specific T and B cells. Thus, to determine to what extent the membrane-displayed, prefusion-stabilized ConCv5 KIKO* Env trimer elicits such T and B cell responses, we analyzed the Env-specific responses induced in mice when ConCv5 KIKO* was delivered in homologous or heterologous prime/boost vector combinations (VSV-GP, NYVAC, and DNA; groups 1–4) or as recombinant soluble protein (group 5). In addition, the recombinant protein was added as late booster to enhance the humoral response in all groups. For this, groups of BALB/c mice (n = 10) were immunized at weeks 0 and 4 with homologous or heterologous combinations of VSV-GP-ConCv5 KIKO*, NYVAC-ConCv5 KIKO*, or DNA-ConCv5 KIKO* vectors or with the ConCv5 KIKO* prefusion-stabilized Env trimer protein + MPLA by i.m. route as described in Materials and Methods. At 10 days post-boost, five animals of each group were sacrificed to analyze both cellular and humoral acute immune responses against HIV-1 ConCv5 KIKO* protein. At weeks 8 and 12, the remaining mice received two additional protein boosts by i.m. route, and at 90 days post-second protein boost, animals were sacrificed. HIV-1 ConCv5 Env-specific T cells, Tfh cells, and GC B cells and humoral responses were determined at 10 days post-boost (to analyze the effect of VSV-GP, NYVAC, or DNA vectors alone) and at 90 days post-second protein boost (to analyze the effect of the Env component and the durability of the response). Blood was also collected by submandibular bleeding at 10 days post-second protein boost to determine the humoral HIV-1 ConCv5 KIKO*-specific acute responses. The immunization schedule, the timing for the analysis of humoral and cellular responses, and the different immunization groups are shown in **Figure 3**.

#### 3.4.1 HIV-1 Env consensus C-specific cellular immune responses at 10 days post-boost (effect of VSV-GP, NYVAC, or DNA vector delivery alone)

##### 3.4.1.1 HIV-1 Env-specific T cell responses

First, we analyzed the HIV-1 Env consensus C-specific T cells elicited by the different combinations of VSV-GP-ConCv5 KIKO*, NYVAC-ConCv5 KIKO*, and DNA-ConCv5 KIKO* vectors or recombinant ConCv5 KIKO* prefusion-stabilized Env trimer at 10 days post-boost as indicated in **Figure 3**. Splenocytes from immunized mice were stimulated *ex vivo* for 6 h with the HIV-1 gp160 (consensus C) peptide pool, and after the stimulation period, cells were incubated with specific antibodies for surface markers to identify T cell lineage (CD3, CD4, and CD8) and for effector cytokines (IFN-γ, IL-2, and TNF-α) and degranulation (CD107a) to establish responding cells. The HIV-1 Env consensus C-specific T cell responses were defined by the percentage of T cells with CD4 (single/live/CD3^+^/CD4^+^) or CD8 (single/live/CD3^+^/CD8^+^) phenotype that produced IFN-γ and/or IL-2 and/or TNF-α or by the percentage of T cells with CD4 or CD8 phenotype that expressed CD107a.

**Figure 4:**
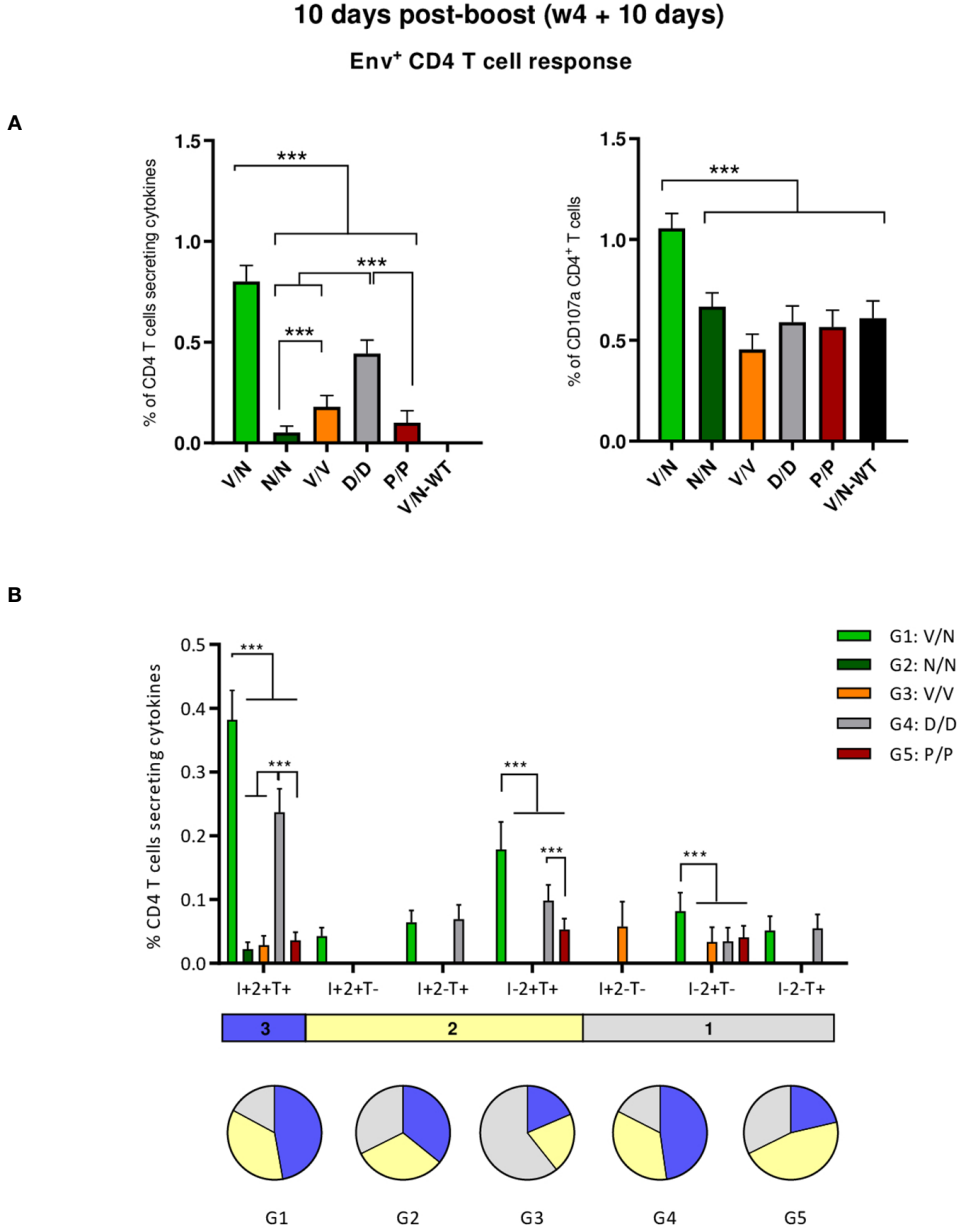
HIV-1 Env-specific T cell responses induced in the spleen of immunized mice at 10 days post-boost. **(A)** Magnitude of the HIV-1 Env-specific CD4 T cell responses induced in the spleens of immunized mice measured at 10 days post-boost by Intracellular Cytokine Staining (ICS) assay after stimulation of pooled splenocytes of each group with HIV-1 gp160 (consensus C) peptide pool. Left: the total value in each group indicates the sum of the percentages of CD4^+^ T cells producing IFN-γ and/or TNF-α and/or IL-2 after stimulation with the HIV-1 gp160 (consensus C) peptide pool. Right: The total value in each group represents the percentages of CD4^+^ T cells expressing CD107a after stimulation with the HIV-1 gp160 (consensus C) peptide pool. All data are background-subtracted. 95% confidence interval (CI) is shown. ***p < 0.001. **(B)** Polyfunctional profile of the HIV-1 Env-specific CD4 T cells in the different immunization groups. The positive combinations of the responses are indicated on the *x*-axis, while the percentages of the functionally different cell populations within the total CD4 T cells are represented on the *y*-axis. Specific responses are grouped and color-coded based on the number of functions. I, IFN-γ; 2, IL-2; T, TNF-α. All data are background-subtracted. 95% CI is shown. ***p < 0.001.

As shown in the left panel of **Figure 4A**, the combination of VSV-GP-ConCv5 KIKO* in the prime and NYVAC-ConCv5 KIKO* in the boost (V/N) induced the highest HIV-1 Env-specific CD4 T cell response (*p* < 0.001), determined by the percentage of cytokine-producing CD4 T cells, followed by the homologous combination of DNA-ConCv5 KIKO* (D/D) (*p* < 0.001). Regarding CD107a expression as an indirect degranulation marker, the combination V/N was the only immunization regimen that induced HIV-1 Env-specific CD4 T cells exceeding the background level (*p* < 0.001) (**Figure 4A**, right panel). No specific CD8^+^ T cell response above RPMI levels was detected in immunized mice, correlating with the previously reported preferential activation of CD4^+^ T cells by the NYVAC vector (51–53).

We also determined the quality of the HIV-1 Env-specific CD4 T cell responses by the pattern of cytokine production by activated T cells. Based on the analysis of IFN-γ, IL-2, and TNF-α, seven different HIV-1 Env-specific CD4 T cell populations were identified (**Figure 4B**). Vaccine-induced CD4 T cell responses were highly polyfunctional in all immunization groups, except in group 3 (V/V), with more than 80% of CD4^+^ T cells exhibiting two or three functions in groups 1 (V/N) and 4 (D/D) and more than 65% in groups 2 (N/N) and 5 (P/P). These data correlated with the magnitude of the HIV-1 Env-specific CD4 T cell response since the groups with the highest Env-specific CD4 T cell responses (V/N and D/D) exhibited the highest polyfunctional profile with 48% of CD4^+^ T cells producing the three cytokines assayed, whereas 35% produced two cytokines and 17% produced only one. CD4 T cells producing simultaneously IFN-γ + IL-2 + TNF-α followed by IL-2 + TNF-α were the most representative populations, although the absolute frequencies of these populations were significantly higher in the V/N and D/D groups (**Figure 4B**).

##### 3.4.1.2 HIV-1 Env-specific Tfh cell responses

CD4 Tfh cells are essential for the development and conservation of GC reactions, a critical event that leads to the generation of long-lived high-affinity antibodies. This cooperation between Tfh and B cells is mediated by different cell-associated and soluble factors, including CD154 (CD40L), IL-21, IL-10, IL-4, and ICOS (40). Since the quality and frequency of HIV-1-specific Tfh cells have been previously correlated with the development of bnAbs (54, 55), we next characterized the induction of this cellular subpopulation by the different prime/boost immunization protocols. For this, splenocytes or lymphocytes from DLNs were isolated at 10 days post-boost, non-stimulated (RPMI), or stimulated *ex vivo* for 6 h with the HIV-1 gp160 (consensus C) peptide pool and evaluated by flow cytometry as previously described (22). Briefly, lymphocytes were gated on singlets followed by live cell selection. Next, CD4^+^CD8^−^ cells were gated and evaluated based on the expression of PD1 and CXCR5 markers. Total Tfh cells were defined by the double-positive CXCR5^+^PD1^+^ population, and HIV-1 Env-specific Tfh cells were established by the percentage of total CXCR5^+^PD1^+^ cells that produce IL-4 and/or IFN-γ and/or expressed CD40L.

**Figure 5:**
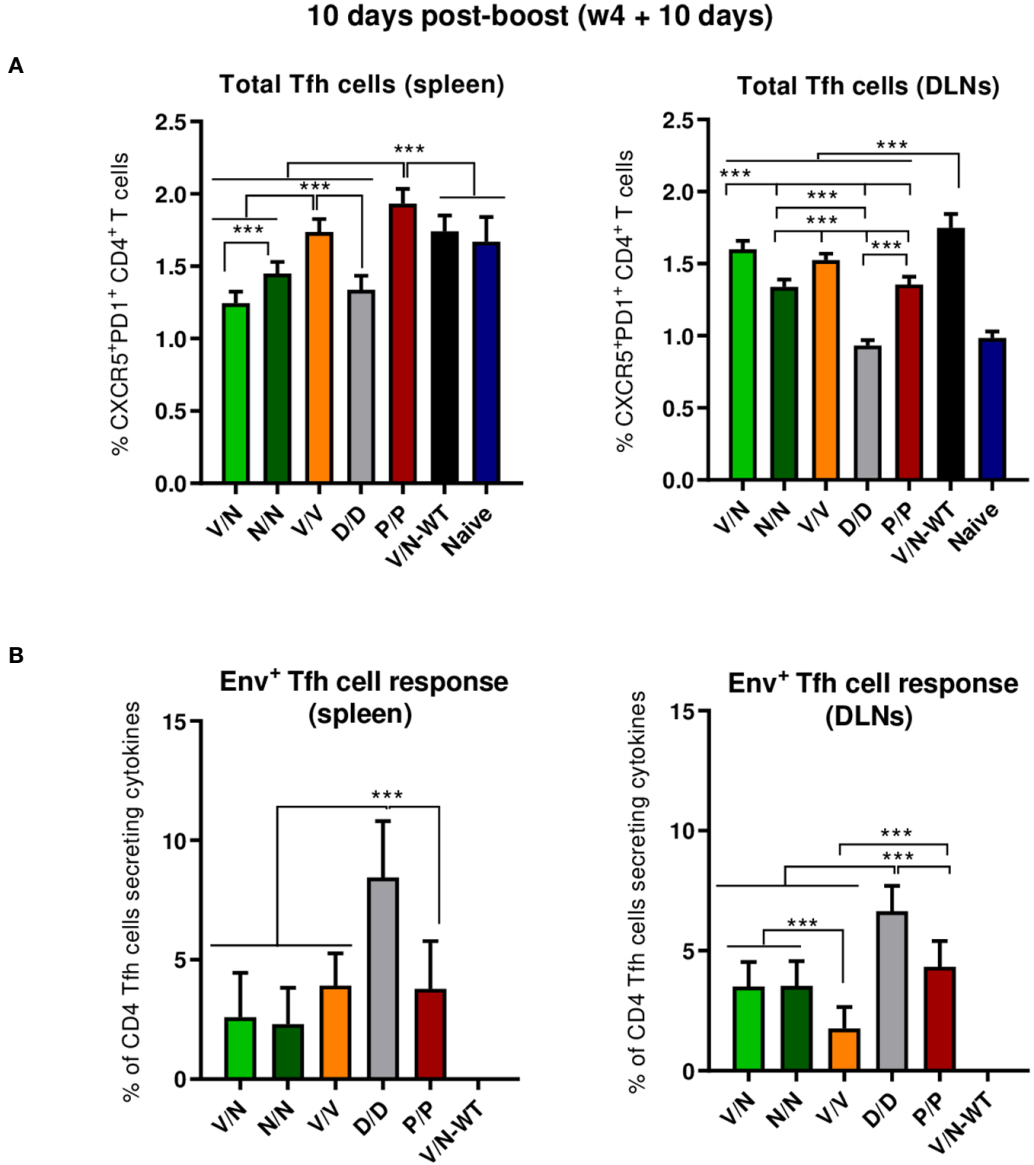
Total and HIV-1 Env-specific Tfh cell responses induced in the spleen and DLNs of immunized mice at 10 days post-boost. **(A)** Magnitude of the total CD4 T cells with Tfh phenotype (CXCR5^+^PD1^+^) in spleen (left) and draining lymph nodes (DLNs) (right) measured by Intracellular Cytokine Staining (ICS) assay in non-stimulated [Roswell Park Memorial Institute (RPMI)] pooled lymphocytes. 95% CI is shown. ***p < 0.001. **(B)** Percentage of the total Tfh cells that are HIV-1 Env-specific in spleen (left) and DLNs (right) measured by ICS following stimulation of pooled lymphocytes with the HIV-1 gp160 (consensus C) peptide pool. The total value in each group represents the sum of the percentages of Tfh^+^ cells producing IL-4 and/or IFN-γ and/or expressing CD154 (CD40L) after stimulation with the HIV-1 gp160 (consensus C) peptide pool. Data are background-subtracted. 95% CI is shown. ***p < 0.001.

As shown in **Figure 5A** (left panel), the homologous P/P combination induced higher levels of total CD4 T cells with Tfh phenotype (CXCR5^+^PD1^+^) in the spleen than the level detected in naïve mice. In DLNs, all the different prime/boost combinations, except the D/D regimen, induced higher levels of total Tfh cells than the level observed in naïve mice (**Figure 5A**, right panel), and some differences were observed between groups with the heterologous combination (V/N-WT) inducing the highest percentage of total Tfh cells (*p* < 0.001), followed by the V/N and V/V regimens. Next, we evaluated the percentage of the HIV-1 Env-specific Tfh cells by quantifying the number of Tfh cells that expressed CD154 (CD40L) and/or produced IFN-γ and/or IL-4 after Env peptide pool stimulation compared to non-stimulated (RPMI) cells. As shown in **Figure 5B**, the homologous combination D/D elicited the highest HIV-1 Env-specific Tfh cell response in the spleen and DLNs (*p* < 0.001).

##### 3.4.1.3 HIV-1 ConCv5 KIKO*-specific GC B cell responses

B cell follicles contain secondary lymphoid structures called GCs in which B cells suffer affinity maturation and class-switch recombination to develop high-affinity antibodies (56–59). Thus, we next evaluated the GC B cell population elicited by the different immunization regimens in the spleen and DLNs at 10 days post-boost by flow cytometry. The gating strategy employed for the identification of GC B cells has been previously reported (22). Briefly, after gating on singlets/lymphocytes/live/CD3^−^CD19^+^ cells, B cells were defined as B220^+^ cells, and from these, GC B cells were identified as GL7^+^CD38^−^ cells. Biotinylated ConCv5 KIKO* protein was used to define the HIV-1 Env-specific GC B cells.

**Figure 6:**
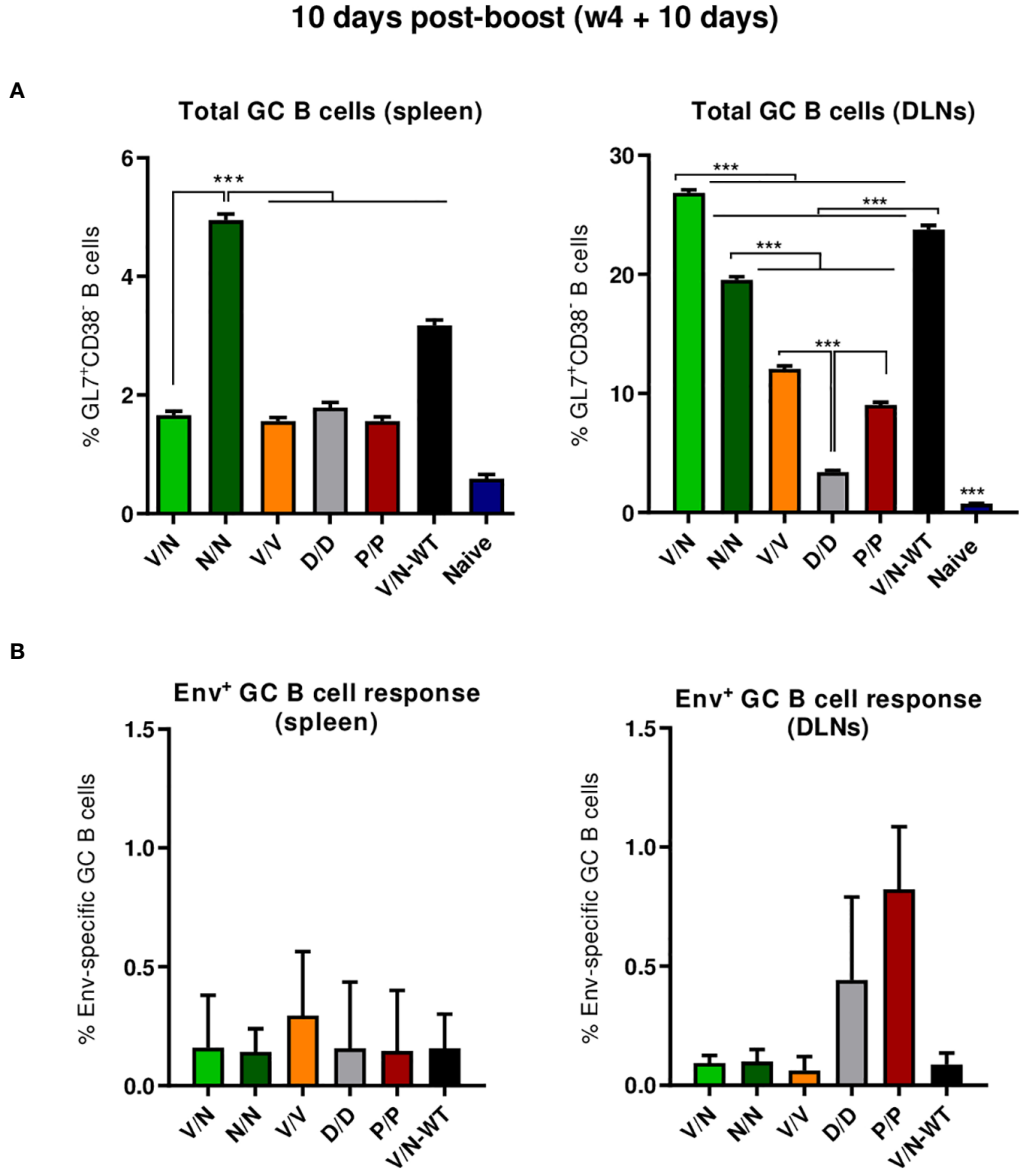
Total and HIV-1 ConCv5 KIKO*-specific GC B cell responses induced in the spleen and DLNs of immunized mice at 10 days post-boost. **(A)** Magnitude of the total germinal center (GC) B cells (GL7^+^CD38^−^) in spleen (left) and draining lymph nodes (DLNs) (right) measured at 10 days post-boost by Intracellular Cytokine Staining (ICS) in pooled lymphocytes from immunized animals. 95% CI is shown. ***p < 0.001. **(B)** Magnitude of the HIV-1 ConCv5 KIKO*-specific GC B cells of the total GC B cells in spleen (left) and DLNs (right) determined at 10 days post-boost by ICS after staining of isolated lymphocytes with biotinylated ConCv5 KIKO* protein. 95% CI is represented.

As shown in **Figure 6A** (left panel), the immunization with the different vector combinations induced high levels of total GC B cells in the spleen compared to the 0.59% of total GC B cells observed in naïve mice. The homologous combination of NYVAC-ConCv5 KIKO* (N/N) induced the highest percentage of total GC B cells (*p* < 0.001), followed by the V/N-WT regimen. In DLNs (**Figure 6A**, right panel), the different prime/boost combinations induced higher levels of total GC B cells than the levels detected in the spleen and also higher levels compared to the 0.73% of total GC B cells observed in the DLNs of naïve mice. The highest percentage of total GC B cells (*p* < 0.001) was observed in the group that received the V/N prime/boost combination followed by the V/N-WT and N/N regimens. No significant differences regarding ConCv5 KIKO*-specific GC B cells of the total GC B cells were noted among the different groups (**Figure 6B**), although a trend to a higher proportion of Env-specific GC B cells in the homologous V/V group in the spleen (**Figure 6B**, left panel) and in the homologous P/P and D/D immunization groups in DLNs (**Figure 6B**, right panel) was observed.

In summary, at the acute phase of the immune response (10 days post-boost), i) the combination VSV-GP-ConCv5 KIKO*/NYVAC-ConCv5 KIKO* induced the highest levels of polyfunctional HIV-1 Env-specific CD4 T cells, ii) the homologous DNA-ConCv5 KIKO* immunization regimen induced the highest levels of HIV-1 Env-specific Tfh cells in the spleen and DLNs, and iii) a trend toward higher levels of ConCv5 KIKO*-specific GC B cells was observed in the homologous VSV-GP-ConCv5 KIKO* immunization group in the spleen and the homologous Protein/Protein or DNA/DNA immunization groups in DLNs.

#### 3.4.2 HIV-1 Env consensus C-specific cellular immune responses at 90 days post-second protein boost (effect of the Env component and durability of the response)

##### 3.4.2.1 Durability of the HIV-1 Env-specific T cell responses

To evaluate the booster effect of ConCv5 KIKO* protein administration and the durability of the Env-specific T cell responses induced by the different vector combinations at 90 days post-second protein boost, splenocytes from immunized animals were stimulated and analyzed as described above.

**Figure 7:**
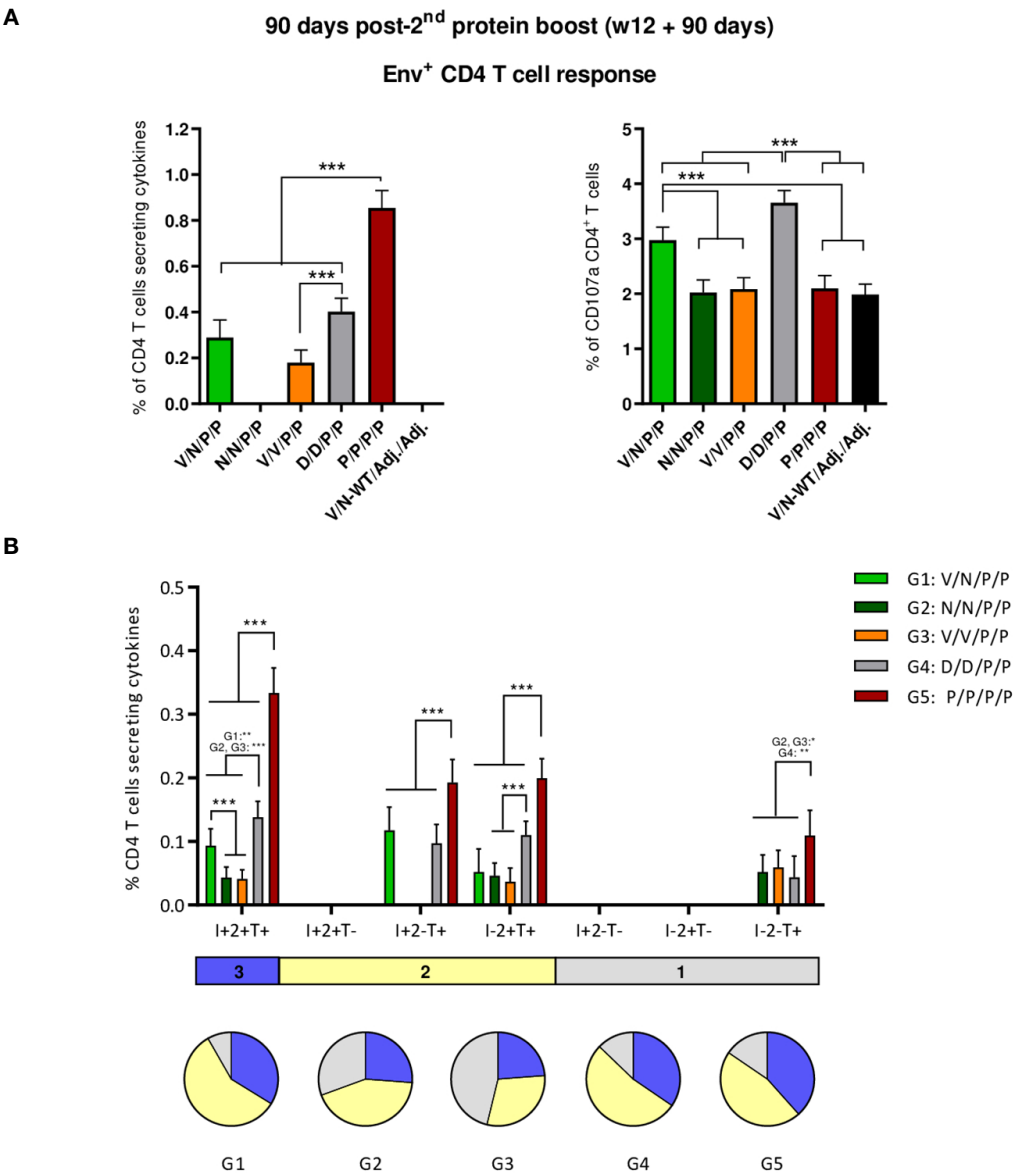
HIV-1 Env-specific T cell responses induced in the spleen of immunized mice at 90 days post-second protein boost. **(A)** Magnitude of the HIV-1 Env-specific CD4 T cell responses measured by Intracellular Cytokine Staining (ICS) assay following stimulation of pooled splenocytes of each group with HIV-1 gp160 (consensus C) peptide pool. Left: **t**he total value in each group represents the sum of the percentages of CD4^+^ T cells producing IFN-γ and/or IL-2 and/or TNF-α after stimulation with the HIV-1 gp160 (consensus C) peptide pool. Right: the total value in each group indicates the percentages of CD4^+^ T cells expressing CD107a after stimulation with the HIV-1 gp160 (consensus C) peptide pool. All data are background-subtracted. 95% CI is shown. ***p < 0.001. **(B)** Polyfunctional profile of the HIV-1 Env-specific CD4 T cells in the different immunization groups. The positive combinations of the responses are indicated on the *x*-axis, while the percentages of the functionally different cell populations within the total CD4 T cells are represented on the *y*-axis. Specific responses are grouped and color-coded based on the number of functions. I, IFN-γ; 2, IL-2; T, TNF-α. All data are background-subtracted. 95% CI is shown. *p < 0.05; **p < 0.005; ***p < 0.001.

As shown in **Figure 7A** (left panel), the protein-only immunization regimen (P/P/P/P) induced the highest levels of HIV-1 Env-specific CD4 T cells (*p* < 0.001), as determined by the percentage of CD4 T cells that produced IFN-γ and/or TNF-α and/or IL-2 followed by the D/D/P/P and V/N/P/P regimens. Regarding CD107a expression, D/D/P/P and the heterologous combination V/N/P/P were the only immunization regimens that induced levels of HIV-1 Env-specific CD4 T cells exceeding the background level (*p* < 0.001) (**Figure 7A**, right panel). As observed during the acute phase (10 days post-boost), no specific CD8 T cell response above RPMI levels was detected.

Next, we evaluated the polyfunctional profile of the vaccine-induced CD4 T cell responses by the pattern of cytokine production (**Figure 7B**). Env-specific CD4 T cell responses were highly polyfunctional in all immunization groups, except in group 3 (V/V/P/P), with more than 80% of CD4 T cells exhibiting two or three functions in groups 1 (V/N/P/P), 4 (D/D/P/P), and 5 (P/P/P/P) and more than 65% in group 2 (N/N/P/P). Again, these data correlated with the magnitude of the Env-specific CD4 T cell response since the groups with the highest HIV-1 Env-specific CD4 T cell responses (P/P/P/P, D/D/P/P, and V/N/P/P) exhibited the highest polyfunctional profile with 36% of CD4^+^ T cells producing three cytokines, 52% producing two cytokines, and 12% producing only one. CD4 T cells producing simultaneously IFN-γ + IL-2 + TNF-α followed by IL-2 + TNF-α, IFN-γ + TNF-α, and TNF-α were the vaccine-induced activated populations, although the absolute frequencies of these populations were significantly higher in the P/P/P/P, D/D/P/P, and V/N/P/P groups (**Figure 7B**).

##### 3.4.2.2 Durability of the HIV-1 Env-specific Tfh cell responses

We next characterized the HIV-1 Env-specific Tfh cell responses induced by the different immunization regimens at 90 days post-second protein boost after splenocytes or lymphocytes from DLNs were non-stimulated (RPMI) or stimulated with the HIV-1 gp160 (consensus C) peptide pool.

**Figure 8:**
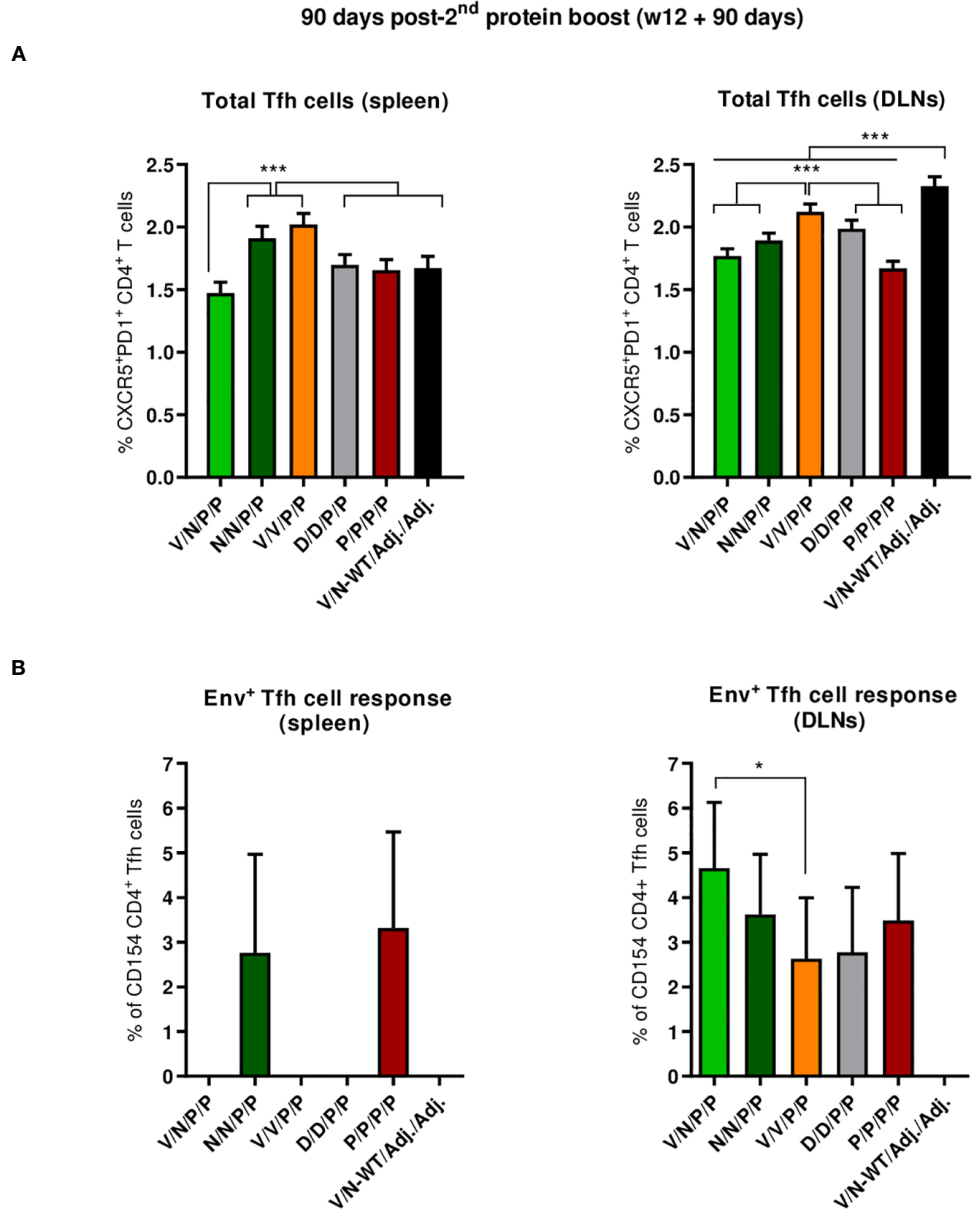
Total and HIV-1 Env-specific Tfh cell responses induced in the spleen and DLNs of immunized mice at 90 days post-second protein boost. **(A)** Magnitude of the total CD4 T cells with Tfh phenotype (CXCR5^+^PD1^+^) in spleen (left) and draining lymph nodes (DLNs) (right) measured by Intracellular Cytokine Staining (ICS) assay in non-stimulated RPMI pooled lymphocytes from immunized mice. 95% CI is shown. ***p < 0.001. **(B)** Percentage of the HIV-1 Env-specific Tfh cells in spleen (left) and DLNs (right) following stimulation of pooled lymphocytes with the HIV-1 gp160 (consensus C) peptide pool. The total value in each group represents the sum of the percentages of Tfh^+^ cells producing IL-4 and/or IFN-γ and/or expressing CD154 (CD40L) following stimulation with the HIV-1 gp160 (consensus C) peptide pool. Data are background-subtracted. 95% CI is shown. *p < 0.05.

The combinations V/V/P/P and N/N/P/P induced the highest levels of total Tfh cells in the spleen (*p* < 0.001) (**Figure 8A**, left panel), while in DLNs, the combinations V/N-WT/Adj./Adj., followed by the V/V/P/P regimen, induced the highest levels of total Tfh cells (*p* < 0.001) (**Figure 8A**, right panel). The percentage of HIV-1 Env-specific Tfh cells was determined by quantifying the number of Tfh cells that expressed CD154 (CD40L) and/or produced IFN-γ and/or IL-4 after Env peptide pool stimulation compared to non-stimulated cells (RPMI). As shown in the left panel of **Figure 8B**, the regimens P/P/P/P and N/N/P/P were the only ones that induced HIV-1 Env-specific Tfh cell response in the spleen. In DLNs (**Figure 8B**, right panel), all the different prime/boost combinations induced HIV-1 Env-specific Tfh cells compared to the control group (V/N-WT/Adj./Adj.), and some differences were observed between groups, with the heterologous combination V/N/P/P inducing the highest HIV-1 Env-specific Tfh cell response (*p* < 0.05).

Overall, at the memory phase of the immune response (90 days post-second protein boost and 6 months after initiation of the study), i) the protein-only immunization regimen (P/P/P/P), the homologous combination D/D/P/P, and the heterologous combination V/N/P/P induced the highest polyfunctional HIV-1 Env-specific CD4 T cell responses; ii) HIV-1 Env-specific Tfh cells were induced, with specific induction by the combinations P/P/P/P and N/N/P/P in the spleen and a higher induction by the combination V/N/P/P in DLNs.

#### 3.4.3 HIV-1 ConCv5 KIKO*-specific humoral immune responses induced by the different vector combinations during the study

An essential requirement for vector combinations used as vaccine candidates is their ability to induce not only HIV-1-specific cellular immune responses but also high levels of durable HIV-1-specific antibodies. For this reason, we first analyzed the humoral responses induced by the different combinations of VSV-GP-ConCv5 KIKO*, NYVAC-ConCv5 KIKO*, and DNA-ConCv5 KIKO* vectors or prefusion-stabilized ConCv5 KIKO* Env trimer protein followed by two consecutive boosts with the same Env trimer. For this, the serum reactivity at 10 days post-boost (effect of VSV-GP, NYVAC, or DNA vectors alone), 10 days post-second protein boost (effect of the Env protein component), or 90 days post-second protein boost (effect of the Env component and durability of the response) against purified prefusion-stabilized ConCv5 KIKO* Env trimer protein was quantified by two approaches, direct and capture ELISA. In addition, the reactivity of the sera toward a diverse panel of Env trimers from HIV-1 clades A, B, and C and the homologous gp120 monomer was assessed.

##### 3.4.3.1 Binding antibody levels in serum against the native homologous ConCv5 KIKO trimer

**Figure 9:**
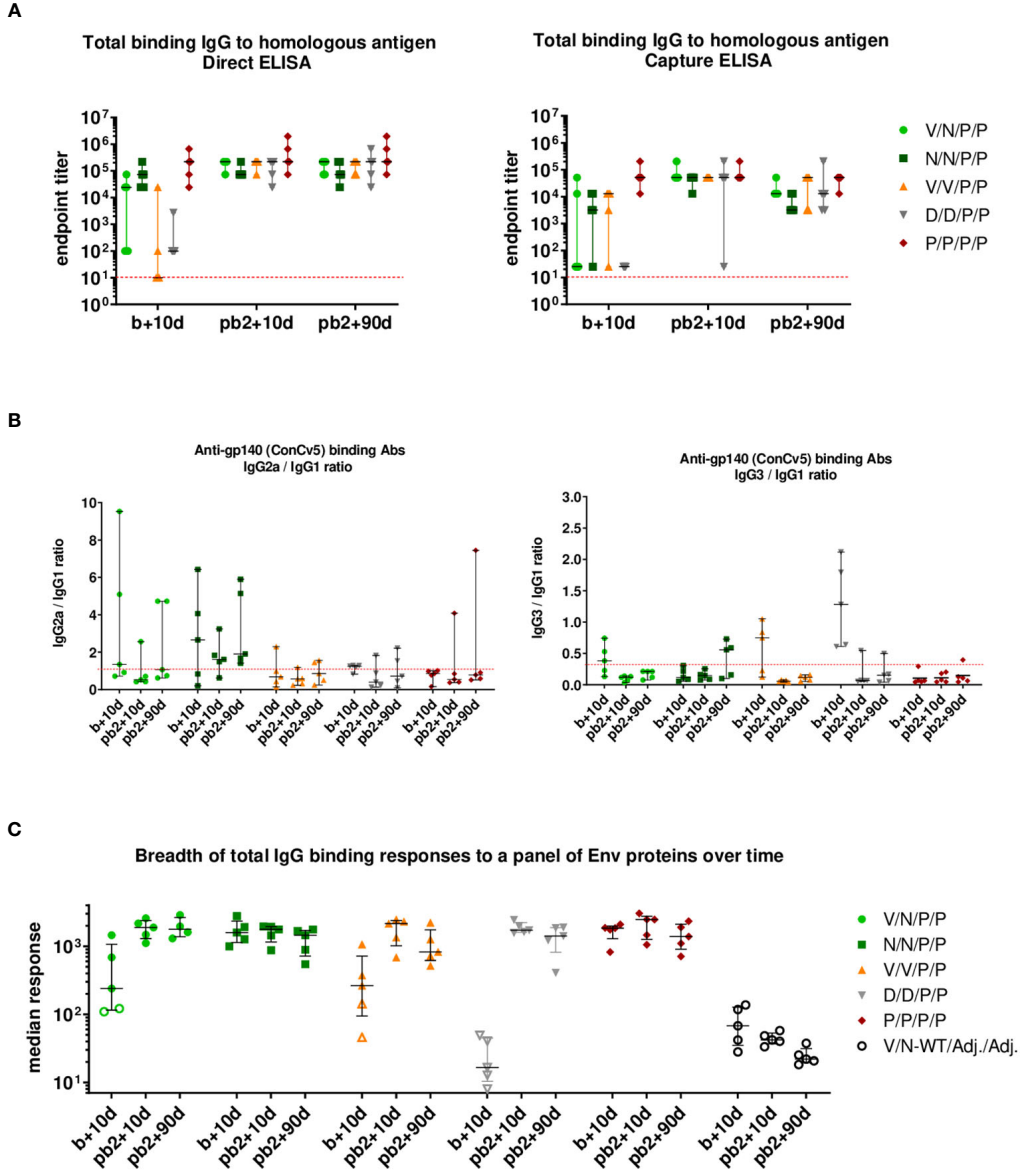
HIV-1 ConCv5 KIKO*-specific humoral responses in the serum of immunized mice during the study. **(A)** Levels of HIV-1 ConCv5 KIKO*-specific total IgG binding antibodies were determined in individual sera from immunized mice at 10 days post-boost (b+10d), 10 days post-second protein boost (pb2 + 10d), or 90 days post-second protein boost (pb2 + 90d) by direct (left) or capture Ni-NTA (right) ELISA as described in Materials and Methods. The endpoint titer represents the last dilution of serum that yielded three times the mean optical density (OD) measured at 450 nm (OD_450_) of the control group. Data for the individual animals as colored forms with median and range are shown. Red dashed line: limit of detection of the assay. **(B)** Levels of HIV-1 ConCv5 KIKO*-specific IgG1, IgG2a, and IgG3 binding antibodies were determined in individual sera from immunized mice at the three time-points indicated as OD_450_ at a serum dilution of 1:8,100 (b+10d) or 1:24,300 (pb2 + 10d, pb2 + 90d) by direct ELISA, and ratios IgG2a/IgG1 (left) or IgG3/IgG1 (right) are represented. Data for the individual animals as colored symbols with median and range are shown. Red dashed line: ratio = 1. **(C)** HIV-1 Env breadth analysis of antibody responses induced by the different immunization regimens. BAMA analysis of sera toward a panel of eight Env variants over the three time-points indicated is shown. Positivity of a response was determined as three times the signal of the control group at the respective time-point. One point represents the average MFI* for eight different Env antigens per mouse. Data for the individual animals are shown as colored symbols with median and range. Non-responders are represented by open symbols.

As shown in the left panel of **Figure 9A** (direct ELISA), the prime/boost immunization regimens P/P and N/N induced high levels of HIV-1 Env-specific total IgG binding antibodies with titers ranging from 10^4^ to 10^6^ at 10 days post-boost (b+10d), while some animals immunized with V/N, V/V, or D/D developed Env-specific total IgG endpoint titers just above the limit of detection of the assay. The two late protein administrations increased the total IgG binding antibody responses in all groups to similar levels to those obtained with the protein-only regimen already after two doses (P/P) (pb2 + 10d). At 90 days post-second protein boost (pb2 + 90d), the levels of HIV-1 Env-specific IgG binding antibodies were maintained in all groups, being similar to those obtained with the two-dose-only protein regimen (P/P). The protein-only regimen (P/P/P/P) induced the highest levels of HIV-1 Env-specific total IgG binding antibodies during the study. At 0.5 to 1 log lower endpoint titers, overall similar patterns were obtained when antibody titers were assessed using Ni-NTA capture ELISA, where the homologous close-to-native prefusion-stabilized ConCv5 KIKO 473T (SOSIP) gp140 Env protein was captured by Ni-NTA (**Figure 9A**, right panel). Whereas the protein-only regimen (P/P/P/P) could induce titers exceeding 10^4^ in all individuals of the immunization group during the study, the regimens using different combinations of viral vectors led to one or several non-responders or in the case of DNA to no responders at all at 10 days post-boost (b+10d). After the two protein boosts (pb2 + 10d), all groups reached comparable median endpoint titers, and the titers of the protein-only group remained at the level of 10 days post-boost. At 90 days post-second protein boost (pb2 + 90d), median responses toward ConCv5 KIKO 473T were sustained with minor declines in some of the groups (**Figure 9A**, right panel).

We next analyzed the IgG subclasses (IgG1, IgG2a, and IgG3) elicited by the different vector combinations and calculated IgG2a/IgG1 or IgG3/IgG1 ratios at the three time-points analyzed. As shown in **Figure 9B**, the immunization protocol N/N/P/P induced an HIV-1 Env-specific response strongly polarized toward the Th1-associated IgG2a subclass. The highest IgG3/IgG1 ratios were observed in the D/D group followed by the V/V immunization group, although this reverted after the late protein boost.

##### 3.4.3.2 Determination of breadth of serum response to a panel of Env proteins

In addition to the homologous titers, we also assessed the breadth of binding responses to a diverse panel of Env proteins (**Figure 9C**). The panel consisted of prefusion-stabilized, closed trimers from clades A (BG505 SOSIP), B (AMC008 SOSIPv4.2 and AMC011 SOSIPv4.2), and C (16055 SOSIP and sC23v4 KIKO 473T); two rather open trimers from clade C (ConCv1 KIKO NFL and 96ZM651 REKS.664); and one clade C gp120 monomer (ConC KIKO). While at 10 days post-boost (b+10d) significant differences occurred between various groups with regard to the breadth of their anti-Env response with single animals being non-responders, all immunization regimens induced comparable median breadth in binding antibody responses at 10 days post-second protein boost (pb2 + 10d), and this breadth of the responses remained roughly constant up to 90 days post-second protein boost (pb2 + 90d) (**Figure 9C**).

In summary, when antibody responses were evaluated, i) the immunization protocols P/P/P/P, N/N/P/P, and V/N/P/P induced high levels of HIV-1 Env-specific total IgG binding antibodies at 10 days post-boost; ii) two doses of protein increased the total IgG binding antibody responses in all groups to similar levels to those obtained with the two-dose-only protein regimen (P/P); iii) at 90 days post-second protein boost, Env-specific binding IgG antibody levels were maintained in all groups, highlighting that these prime/boost regimens tend to increase the durability of humoral responses; iv) the immunization regimen P/P/P/P induced the highest Env-specific total IgG binding antibody responses during the study; v) a preferential Th1-associated IgG2a subclass was elicited by the immunization regimen N/N/P/P; and vi) the breadth of the anti-Env response in the different immunization groups was comparable during the study.

## 4 Discussion

Despite great efforts in the development of HIV-1 vaccine candidates and immunization strategies aimed at conferring long-lasting protective immunity, to date there is no effective vaccine. Multiple prime/boost immunization regimens including different combinations of HIV-1 proteins and vectors to induce broad and polyfunctional HIV-1-specific T and B cell responses have been evaluated in preclinical and clinical trials (16, 19).

In an effort to obtain breadth and durability of the immune responses directed against HIV-1, we generated non-viral (DNA) and viral (VSV-GP and NYVAC) vectors expressing a set of next-generation clade C Env trimers, which were optimized for cell surface display following vector delivery. The membrane-tethered prefusion-stabilized gp140:GΔ6 ConCv5 Env trimer turned out to be the best-in-class candidate from all tested constructs, demonstrating excellent antigenicity regarding binding of selected bnAbs, whereas epitopes known to be preferentially recognized by nnAbs were less accessible. Next, the impact of vector-mediated delivery of gp140:GΔ6 ConCv5 on the priming of T and B cell responses was determined in mice. We demonstrated that the combination of VSV-GP-ConCv5 KIKO* in the prime and NYVAC-ConCv5 KIKO* in the boost is the most effective regimen to induce HIV-1 Env-specific CD4 T cell responses in the absence of a protein boost. After HIV-1 ConCv5 KIKO* protein administration, the T cell activation levels were maintained between groups. When antibody responses were evaluated after protein boosts, the different vector combinations elicited high levels of HIV-1 Env-specific total IgG binding antibodies that were similar to the levels induced by the two-dose P/P immunization regimen and remained high during the memory phase, highlighting that the late protein boosts increase the durability of the humoral responses. The immunization regimen N/N/P/P induced preferentially Th1-associated IgG2a subclass; in addition, the breadth of the anti-Env response observed in the different immunization groups was comparable during the study.

The HIV-1 clade C membrane-tethered sC23v4 KIKO* and ConCv5 KIKO* envelopes were designed to form close-to-native trimers at the cell surface with the aim of inducing potent Env-specific antibody responses. The close-to-native conformation of the two membrane-displayed Env trimers delivered by three different vectors (DNA, VSV-GP, or NYVAC) was confirmed by flow cytometry through the analysis of the binding profile of bnAbs and nnAbs. The differential Ab recognition profiles observed for both membrane-tethered Env trimers, as exemplified by bnAbs targeting V1/V2 quaternary epitopes, indicate that slightly different conformations are adopted by both membrane-tethered Envs, with ConCv5 KIKO* showing a more closed and native-like conformation than sC23v4 KIKO*. Therefore, due to its superior bnAb recognition profile, ConCv5 KIKO* was selected for immunological analysis following i) vector delivery and ii) after two booster immunizations with the corresponding prefusion-stabilized Env trimer protein.

It is widely accepted that HIV-1 vaccine candidates should harness both humoral and cellular immune responses. For example, delivery of antigens from simian immunodeficiency virus (SIV) by a rhesus cytomegalovirus vector has been reported to elicit a broad memory T cell response able to inhibit SIV replication in several macaques early after infection and to prevent the establishment of a latent reservoir (60). Polyfunctional CD4^+^ T cells have also been reported to play a role in antibody-mediated vaccine efficacy in RV144 vaccinees (13). In this regard, we showed that the combination VSV-GP-ConCv5 KIKO*/NYVAC-ConCv5 KIKO* is the most effective immunization protocol to activate HIV-1 Env-specific highly polyfunctional CD4 T cells at 10 days post-boost (in the absence of protein boost), confirming previous results from our group (22). At the memory phase, the protein-only immunization regimen (P/P/P/P), the homologous combination of DNA-ConCv5 KIKO* prime and protein boost (D/D/P/P), and the heterologous combination V/N/P/P induced the highest levels of polyfunctional HIV-1 Env-specific CD4 T cells, indicating a modulating effect of the protein component over the T cell response elicited.

The development of HIV-1 Env-specific bnAbs requires extensive somatic hypermutation (SHM) (61), and cooperation between Tfh and GC B cells is responsible for the maturation of antibody affinity (62, 63). Preclinical and clinical assays have reported that the frequency of Tfh and GC B cells correlated with the development of bnAbs (64–66). Furthermore, a subpopulation of highly functional blood-circulating memory Tfh cells has been shown to support the development of HIV-1-specific bnAbs in HIV-1-infected individuals (65), and a strong correlation between the early maintenance of a specific subpopulation of peripheral Tfh cells and the breadth of the nAbs during chronic HIV-1 infection has been reported (67), suggesting a critical role of the peripheral Tfh cells in the primary activation of B cells needed for affinity maturation, SHM, and generation of bnAbs. Higher percentages of resting memory Tfh cells in HIV-1-infected individuals with plasma bnAbs compared to HIV-1-infected individuals without bnAbs (68) have also been reported. In summary, all these observations support the design of novel vaccine candidates and/or immunization regimens able to elicit improved GC and Tfh responses and, consequently, high levels of SHM that eventually may result in the development of HIV-1 bnAbs (54).

When we evaluated the HIV-1 Env-specific Tfh cells at 10 days post-boost, the homologous combination DNA-ConCv5 KIKO* (D/D) induced the highest levels of Env-specific Tfh cells in the spleen and DLNs. In addition, we detected HIV-1 Env-specific GC B cells in the homologous V/V immunization regimen in the spleen and homologous P/P and D/D immunization groups in DLNs. At the memory phase, the combinations P/P/P/P and N/N/P/P were the only immunization regimens that induced HIV-1 Env-specific Tfh cells in the spleen, while in DLNs, the heterologous combination V/N/P/P induced the highest levels of HIV-1 Env-specific Tfh cells.

Another important finding is the induction of high and durable levels of HIV-1 Env-specific binding antibodies by the different immunization groups. In particular, the immunization regimens P/P/P/P, N/N/P/P, and V/N/P/P elicited high levels of HIV-1 Env-specific total IgG binding antibodies (endpoint titers from ELISA ranging from 10^4^ to 10^6^) in the absence of Env protein component (10 days post-boost). However, the protein-only group (P/P) induced the highest titers with 100% responding animals already at this time-point. The two protein boosts increased the total IgG binding antibody responses in all groups to levels comparable to those obtained with the two-dose-only protein regimen (P/P), with the immunization regimen P/P/P/P inducing the highest Env-specific total IgG binding antibody responses. At late times (90 days post-second protein boost), the levels of HIV-1 Env-specific binding IgG antibodies were nearly maintained in all groups being similar—but with higher variation between the animals—to those observed with the protein-only regimen, highlighting that these prime/boost immunization regimens elicit long-lasting HIV-1 Env-specific humoral responses. Moreover, the immunization regimen N/N/P/P induced a polarized HIV-1 Env-specific response toward the Th1-associated IgG2a subclass. This Th1 polarization has been previously reported with similar immunization regimens, although the HIV-1 clade C gp145 (96ZM651) protein showed an inferior bnAb recognition profile when used as an antigen (22). Finally, the breadth of the Env-specific response was comparable between the different immunization groups in the study, suggesting that the breadth of binding antibodies is determined by the antigen *per se* rather than by the delivery modality and schedule. Although these data suggest that two or more immunizations with the Env protein only might be sufficient to induce high titer antibody responses, it is widely accepted that an HIV-1 vaccine should activate not only the humoral response with induction of bnAbs but also other antibody-mediated effector functions such as ADCC and ADCVI, as well as cellular responses with activation of specific CD4^+^ helper and CD8^+^ cytotoxic T cells. In this context, vector-mediated antigen delivery has been demonstrated to be an essential prerequisite for the priming and maintenance of CD4^+^ and CD8^+^ T cell responses (23, 60), and HIV-1-specific T cells induced by vector-mediated immunization regimens can also contribute to the protective immune profile and vaccine efficacy observed in different clinical trials (13, 69, 70). In this sense, non-viral (DNA) and viral (VSV-GP, NYVAC) vectors and their combinations used in the present work have the additional capacity to enhance the HIV-1 Env-specific cellular responses.

The induction of virus-specific bnAbs is the essential immune correlate of protection for most of the successful vaccines based on viral vectors (51, 71, 72). For HIV-1, bnAbs have been described to target different regions of the Env trimer including the variable loops 1 and 2 (V1/V2) glycans, the CD4 binding site, and the V3 glycan on the gp120 protein, as well as the gp120/gp41 interface (73). The design of immunogens and vaccination regimens with the capacity to induce bnAbs able to neutralize tier 2 envelope proteins is a main goal in the vaccine field (74, 75). These efforts are based on the use of structural antibody and Env information, and in particular epitope recognition, to combine relevant epitopes in new immunogens with the aim of selectively inducing a desired bnAb response. However, although bnAbs exhibit a potent neutralization capacity *in vitro* and have shown promising results as prophylactic and therapeutic biologic agents (76–79), they are very difficult to induce by vaccination (80). bnAbs are commonly isolated after years of HIV-1 infection, do not usually protect the individuals who develop them, and show mostly unusually high levels of SHM and frequently long complementarity-determining regions (81). In the only modestly protective RV144 clinical trial, only antibodies able to weakly neutralize tier 1 viruses were detected after vaccination (5, 82). Instead, evaluation of correlates of protection indicates a potential role for nnAbs toward the variable loops 1–2 (V1/V2), likely via antibody-dependent effector functions (12, 83, 84), and Fc-mediated recruitment of effector cells.

Although great efforts have been devoted to the development of optimized Env antigens aiming toward mimicking the close-to-native prefusion Env trimer, only limited tier 2 neutralization breadth has been accomplished to date (85–88). These observations inspire a more holistic multifactorial approach for antibody protection induced by vaccination that could include non-neutralizing but broadly reactive and functional Abs and bnAbs. In this work, the flow cytometry-based bnAb-binding assay performed in transfected or infected cells showed favorable binding profiles toward the membrane-displayed ConCv5 KIKO*, especially regarding the binding to the CD4 binding site (VRC01) and the V1/V2 quaternary structure epitopes (PGT145). This observation strongly supports the assumption of a closed and native-like conformation of membrane-tethered ConCv5 KIKO* Env trimer, which is the desired property for a candidate immunogen.

The analysis of neutralizing activity in mouse serum samples is hard to achieve due to the small volumes of serum obtained, the known high background already in pre-immune serum, and the limited mouse antibody repertoire. However, studies in rabbits have reported the induction of tier 1A neutralizing antibodies following immunization with VSV-GP-based vaccine candidates expressing first-generation, non-prefusion-stabilized trimeric Envs (21). Preclinical studies in rabbits and NHPs are underway to determine the capacity of the herein-described prefusion-stabilized, close-to-native Env trimers to induce tier 2 neutralizing antibody responses. Ongoing NHP studies will also shed light on the potential impact of a heterologous vector prime preceding booster immunizations with the prefusion-stabilized trimers on neutralization potency, durability, and breadth, as well as other antibody-mediated effector functions (e.g., ADCC and ADCVI) and also Env-specific CD4 and CD8 T cell responses.

Relying on delivery modalities presented herein and by including an engineered ConCv5 derivative (ConCv5-GT; (89)), which has recently proven to functionally activate recombinant B cells expressing the germline VRC01 B cell receptor as priming immunogen, we will also be able to test the principle of guiding bnAb responses in appropriate human immune system and transgenic mouse models (14, 89–92). Eventually, the novel ConCv5-GT Env trimer together with the ConCv5 KIKO* trimer, both available as GMP grade drug products, and the non-viral (DNA) and viral (VSV-GP and NYVAC) vectors analyzed in this work should be considered as optimized immunogen components against HIV-1 for further clinical trials.

## Data availability statement

The raw data supporting the conclusions of this article will be made available by the authors, without undue reservation.

## Ethics statement

Experimental protocols for the in vivo assays in mice were authorized by the Ethical Committee of Animal Experimentation of Centro Nacional de Biotecnología (CEEA-CNB, Madrid, Spain) in agreement with International EU Guidelines 2010/63/UE on protection of animals for scientific purposes, Spanish National Law 32/2007 on animal welfare, exploitation, transport and sacrifice and Spanish National Royal Decree RD 53/2013 (permit number PROEX 281/16). The study was conducted in accordance with the local legislation and institutional requirements.

## Author contributions

BP: Formal analysis, Investigation, Methodology, Validation, Writing – original draft, Writing – review & editing. AH: Formal analysis, Investigation, Methodology, Validation, Writing – original draft, Writing – review & editing. CG: Formal analysis, Investigation, Methodology, Validation, Writing – review & editing. DP: Investigation, Methodology, Writing – review & editing. ES: Investigation, Methodology, Writing – review & editing. CS: Formal analysis, Writing – review & editing. SW: Investigation, Methodology, Writing – review & editing. MS: Investigation, Methodology, Writing – review & editing. LS: Investigation, Writing – review & editing. BA: Formal analysis, Validation, Writing – review & editing. SD: Visualization, Writing – review & editing. DV: Funding acquisition, Resources, Writing – review & editing. YL: Funding acquisition, Resources, Writing – review & editing. GP: Funding acquisition, Resources, Writing – review & editing. JK: Conceptualization, Formal analysis, Investigation, Methodology, Supervision, Validation, Visualization, Writing – original draft, Writing – review & editing. ME: Conceptualization, Funding acquisition, Investigation, Methodology, Resources, Supervision, Visualization, Writing – original draft, Writing – review & editing. RW: Conceptualization, Funding acquisition, Investigation, Methodology, Supervision, Validation, Visualization, Writing – original draft, Writing – review & editing.
