# Supplementary material for: Potency and durability of T and B cell immune responses after homologous and heterologous vector delivery of a trimer-stabilized, membrane-displayed HIV-1 clade ConC Env protein
Source: Front Immunol. 2023 Nov 17;14:1270908. doi: 10.3389/fimmu.2023.1270908 (PMC10690772; doi:10.3389/fimmu.2023.1270908)
Supplement: Supplementary file 1 [file DataSheet_1.docx]

Supplementary Material

# Supplementary Figures

**SUPPLEMENTARY FIGURE 1. *In vitro* characterization of NYVAC-sC23v4 KIKO* and NYVAC-ConCv5 KIKO* recombinant viruses.** **(A)** Confirmation of the insertion of the *HIV-1 sC23v4 KIKO* and ConCv5 KIKO** genes into NYVAC genome by PCR analysis. DNA was extracted from BSC-40 cells infected at 5 pfu/cell with NYVAC-WT, NYVAC-sC23v4 KIKO* or NYVAC-ConCv5 KIKO* viruses. Primers TK-L and TK-R spanning VACV *J2R* (TK) flanking regions were used for PCR analysis of TK locus. In parental NYVAC, a 423 bp-product was observed, while in NYVAC-sC23v4 KIKO* and NYVAC-ConCv5 KIKO* unique 2725- and 2677-bp products were obtained, respectively. **(B)** Analysis of the virus growth profile of NYVAC-sC23v4 KIKO* and NYVAC-ConCv5 KIKO* viruses in a permissive cell line. Monolayers of primary CEF cells were infected at 0.1 pfu/cell with NYVAC-WT, NYVAC-sC23v4 KIKO* or NYVAC-ConCv5 KIKO* viruses. At different times post-infection (0, 24, 48 and 72 h), infected cells were collected and viral titers in cell lysates were determined by immunostaining plaque assay in BSC-40 cells. As it is observed, the growth kinetics between parental and recombinant viruses were similar in cultured CEF cells, indicating that the insertion of the HIV-1 sC23v4 KIKO* or ConCv5 KIKO* encoding genes into the viral genome does not affect the virus yields. Data points represent titers as mean ± SD of n=2 experiments. **(C)** Time-course expression of HIV-1 gp140:GΔ6 KIKO* proteins in cells infected with NYVAC-ConCv5 KIKO* or NYVAC-sC23v4 KIKO* recombinant viruses by western-blotting. Monolayers of non-permissive HeLa (left panels) or permissive BSC-40 cells (right panels) were infected at 5 pfu/cell with NYVAC-ConCv5 KIKO* (upper panels) or NYVAC-sC23v4 KIKO* (lower panels) recombinant viruses. Cellular pellets (pellet) and supernatants (SN) were collected at different times post-infection (0, 4, 8 and 24 h), fractionated by 8% SDS-PAGE and analyzed by western-blotting using a rabbit polyclonal anti-gp120 antibody to detect the expression of HIV-1 gp140:GΔ6 KIKO* proteins. As it is shown, both HIV-1 gp140:GΔ6 KIKO* proteins were correctly expressed over time in both cell lines with the expression levels in BSC-40 cells (right panels) being higher than in HeLa cells (left panels) due to replication competency in the former cells. Most of Env protein remained cell-associated, as small amounts of protein were detected in cell supernatants. **(D)** Fractionation of viral proteins and localization of the HIV-1 gp140:GΔ6 KIKO* proteins within purified NYVAC virions. Sucrose-purified NYVAC-ConCv5 KIKO* and NYVAC-sC23v4 KIKO* viral preparations were disrupted by sequential detergent treatment and the unfractionated lysate virions (total extract, TE) and the different collected fractions (E1, E2, E3 and C) were analyzed by western-blotting using mouse monoclonal anti-GR3 antibody to detect the presence of HIV-1 gp140:G Δ6 KIKO* proteins. This analysis showed that most of the HIV-1 gp140:GΔ6 KIKO* protein was found in the soluble lipid viral envelope fraction (E1 fraction) in both NYVAC-ConCv5 KIKO* and NYVAC-sC23v4 KIKO* purified preparations, corresponding with the membrane-bound nature of the HIV-1 gp140:GΔ6 KIKO* antigens and indicating that both Env proteins were incorporated within the membrane of the NYVAC virions.

**SUPPLEMENTARY FIGURE 2.** **Correlation of Env-specific signals measured upon expression of Env constructs from the different vectors (DNA, VSV-GP and NYVAC) and antibody staining.** The vaccine vector pVRC8400 and the screening vector pcDNA5-FRT/TO encoding for a set of 11 Env trimer constructs were transiently transfected to HEK293T cells. Likewise, cells were infected with the VSV-GP- or NYVAC-based vectors. Cells were stained with a set of primary anti-Env bnAbs (filled circles) and nnAbs (open circles) at 10 µg/ml. Nonparametric Spearman correlation coefficients were determined using GraphPad Prism Software.

**SUPPLEMENTARY FIGURE 3. Purity and antigenicity of soluble, prefusion-stabilized ConCv5 KIKO 473 (SOSIP) Env trimer (shortly termed ConCv5 KIKO*)**. ConCv5 KIKO* was purified as soluble, trimeric gp140 protein. Purity of the trimer preparation was confirmed by analytical size exclusion chromatography **(A)**, blue native PAGE **(B)** and reducing **(C)** and non-reducing **(D)** SDS-PAGE (amount of protein loaded: 2 µg). **(E)** The antigenicity profile of the ConCv5 KIKO* trimers was assessed compared to its parental trimeric protein ConCv5 KIKO lacking the 473T mutation. Differences in binding affinities (EC50 values) towards the individual antibodies are shown as log2 fold change.

# Supplementary Methods

**1. Characterization of NYVAC-sC23v4 KIKO* and NYVAC-ConCv5 KIKO* Stocks**

**1.1 PCR Analysis of NYVAC-sC23v4 KIKO* and NYVAC-ConCv5 KIKO* Recombinant Viruses**

To analyze the identity and purity of NYVAC-sC23v4 KIKO* and NYVAC-ConCv5 KIKO* viral preparations, DNA was extracted from BSC-40 cells infected with NYVAC-WT, NYVAC-sC23v4 KIKO* or NYVAC-ConCv5 KIKO* viruses at 5 pfu/cell for 24 h as previously described (1). Primers TK-R: 5′-CTGCCGTATCAAGGACA-3′ and TK-L: 5′-TGATTAGTTTGATGCGATTC-3′ spanning VACV TK flanking regions were used for the PCR analysis of TK locus (Supplementary Figure 1A). The amplification reactions were performed with Phusion High-Fidelity DNA polymerase (BioLabs, Ipswich, MA, USA) according to manufacturer's instructions.

**1.2 Analysis of Virus Growth**

To assess the virus growth profile, CEF cells grown in 12-well plates were infected with NYVAC-WT, NYVAC-sC23v4 KIKO* or NYVAC-ConCv5 KIKO* viruses at 0.1 pfu/cell in duplicates. After virus adsorption, the inoculum was removed and infected cells were incubated with fresh DMEM-2% FCS. At different times post-infection (0, 24, 48 and 72 h), cells were collected by scraping (lysates at 5 × 10^5^ cells/mL), freeze-thawed 3 times and briefly sonicated (Supplementary Figure 1B). Virus titers were determined by immunostaining plaque assay in BSC-40 cells as previously described (2).

**1.3 Time-Course Expression of HIV-1 sC23v4 KIKO* and ConCv5 KIKO* Proteins by Western-Blotting Analysis**

To analyze the expression of the HIV-1 sC23v4 KIKO* and ConCv5 KIKO* proteins expressed by NYVAC-sC23v4 KIKO* and NYVAC-ConCv5 KIKO* recombinant viruses, respectively, monolayers of HeLa (non-permissive) or BSC-40 (permissive) cells grown in 24-well plates were infected with NYVAC-WT, NYVAC-sC23v4 KIKO* or NYVAC-ConCv5 KIKO* viruses at 5 pfu/cell. At different times post-infection (0, 4, 8 and 24 h), supernatant and pellet samples were obtained as previously described (3), fractionated by 8% Sodium Dodecyl Sulfate Polyacrylamide Gel Electrophoresis (SDS-PAGE) and analyzed by western-blotting using the rabbit polyclonal anti-gp120 antibody (1:3,000; CNB), followed by goat anti-rabbit-HRP antibody (1:5,000; Sigma-Aldrich, St. Louis, MO, USA) to evaluate the expression of both HIV-1 gp140:GΔ6 KIKO* Env proteins (Supplementary Figure 1C). The immunocomplexes were detected by enhanced chemiluminescence (ECL) system (GE Healthcare, Chicago, IL, USA).

**1.4 Fractionation of the HIV-1 sC23v4 KIKO* and ConCv5 KIKO* Proteins into Different Virion Compartments after Sequential Detergent Treatment of Purified Recombinant NYVAC Particles**

Localization of the HIV-1 gp140:GΔ6 KIKO* Env proteins in NYVAC-sC23v4 KIKO* and NYVAC-ConCv5 KIKO* particles was analyzed by sequential detergent treatment as previously described (4, 5). The presence of the HIV-1 gp140:GΔ6 KIKO* Env proteins in the different fractions (E1, E2, E3 and C) was determined by western-blotting using the mouse monoclonal anti-GR3 antibody (1:500; kindly provided by Sanofi Pasteur), followed by goat anti-mouse-HRP (1:2,000; Sigma-Aldrich) (Supplementary Figure 1D). The immunocomplexes were detected by ECL system (GE Healthcare).

**1.5 Genetic Stability of NYVAC-sC23v4 KIKO* and NYVAC-ConCv5 KIKO* Recombinant Viruses**

To ensure that the HIV-1 gp140:GΔ6 KIKO* Env-encoding genes inserted into the parental NYVAC genome were stably integrated and can be maintained in the viral genome without modifications of the sequence, 7 successive infection passages in CEF cells infected with NYVAC-ConCv5 KIKO* or NYVAC-sC23v4 KIKO* recombinant viruses at low multiplicity of infection were performed. Then, passage 7 was used to infect BSC-40 cells and 26 individual plaques of each virus were picked up and grown in BSC-40 cells. A western-blotting analysis was performed to detect the expression of the HIV-1 gp140:GΔ6 KIKO* Env protein in each individual plaque. 25 out of 26 plaques (96% stability) isolated from passage 7 for both NYVAC-ConCv5 KIKO* and NYVAC-sC23v4 KIKO* recombinant viruses correctly expressed the HIV-1 gp140:GΔ6 KIKO* Env protein, indicating that the insertion of the heterologous genes was highly stable (data not shown).

**2. Antigenicity of Soluble gp140 ConCv5 Env trimers**

Highly pure gp140 trimer preparations of the parental ConCv5 KIKO SOSIP (shortly termed ConCv5 KIKO) as well as ConCv5 KIKO 473T SOSIP (shortly termed ConCv5 KIKO*) were coated on Ni-NTA plates (Qiagen, Hilden, Germany) at 3.5 µg/mL in 100 µL PBS over night at 4°C. Wells were washed three times with Tris-buffered saline (TBS; 150 mM NaCl, 50 mM Tris, pH 7.5) and selected monoclonal antibodies (6) were added in PBS + 2% skim milk in a volume of 50 µL/well in a 4-fold serial dilution starting at 100 nM and incubated for 2 h at RT. Plates were washed six times with TBS and the peroxidase-conjugated rabbit anti-human IgG detection antibody (Agilent, Santa Clara, CA, USA) was added at 1:5,000 in PBS supplemented with 1% (w/v) bovine serum albumin (BSA) and incubated for 1 h at RT. After six washes with TBS, plates were developed using 3,3′,5,5′-tetramethyl-benzidine (TMB) substrate. The reaction was stopped by adding 1 M H_2_SO_4_ and the absorbance at 450 nm was measured on an iMarkTM microplate reader (Bio-Rad, Hercules, CA, USA). EC50 values were determined by applying nonlinear regression using GraphPad Prism (GraphPad Software Inc.).

**REFERENCES**

1. Perdiguero B, Gomez CE, Garcia-Arriaza J, Sanchez-Corzo C, Sorzano COS, Wilmschen S, et al. Heterologous Combination of VSV-GP and NYVAC Vectors Expressing HIV-1 Trimeric gp145 Env as Vaccination Strategy to Induce Balanced B and T Cell Immune Responses. Front Immunol. 2019;10:2941.

2. Ramirez JC, Gherardi MM, Esteban M. Biology of attenuated modified vaccinia virus Ankara recombinant vector in mice: virus fate and activation of B- and T-cell immune responses in comparison with the Western Reserve strain and advantages as a vaccine. J Virol. 2000;74(2):923-33.

3. Perdiguero B, Sanchez-Corzo C, CO SS, Mediavilla P, Saiz L, Esteban M, et al. Induction of Broad and Polyfunctional HIV-1-Specific T Cell Responses by the Multiepitopic Protein TMEP-B Vectored by MVA Virus. Vaccines (Basel). 2019;7(3):57.

4. Gomez CE, Esteban M. Recombinant proteins produced by vaccinia virus vectors can be incorporated within the virion (IMV form) into different compartments. Arch Virol. 2001;146(5):875-92.

5. Perdiguero B, Sanchez-Corzo C, Sorzano COS, Saiz L, Mediavilla P, Esteban M, et al. A Novel MVA-Based HIV Vaccine Candidate (MVA-gp145-GPN) Co-Expressing Clade C Membrane-Bound Trimeric gp145 Env and Gag-Induced Virus-Like Particles (VLPs) Triggered Broad and Multifunctional HIV-1-Specific T Cell and Antibody Responses. Viruses. 2019;11(2):160.

6. Hauser A, Carnell G, Held K, Sulbaran G, Tischbierek N, Rogers L, et al. Stepwise Conformational Stabilization of a HIV-1 Clade C Consensus Envelope Trimer Immunogen Impacts the Profile of Vaccine-Induced Antibody Responses. Vaccines (Basel). 2021;9(7):750.
